# R405W Desmin Knock‐In Mice Highlight Alterations of Mitochondria, Protein Quality Control and Myofibrils in Myofibrillar Myopathy

**DOI:** 10.1002/jcsm.70094

**Published:** 2025-10-30

**Authors:** Sabrina Batonnet‐Pichon, Florence Delort, Alain Lilienbaum, Carolin Berwanger, Dorothea Schultheis, Ursula Schlötzer‐Schrehardt, Andreas Schmidt, Steffen Uebe, Yosra Baiche, Tom J. Eisenack, Débora Broch Trentini, Markus Mallek, Leonid Mill, Ana Ferreiro, Bettina Eberhard, Thomas Lücke, Markus Krüger, Christian Thiel, Rolf Schröder, Christoph S. Clemen

**Affiliations:** ^1^ Basic and Translational Myology, Unit of Functional and Adaptive Biology Université Paris Cité/CNRS UMR 8251 Paris France; ^2^ Institut Cochin Université Paris Cité, INSERM U1016, CNRS Paris France; ^3^ Institute of Aerospace Medicine German Aerospace Center Cologne Germany; ^4^ Institute of Vegetative Physiology, Medical Faculty University of Cologne Cologne Germany; ^5^ Institute of Neuropathology, University Hospital Erlangen Friedrich‐Alexander University Erlangen‐Nürnberg Erlangen Germany; ^6^ Department of Ophthalmology, University Hospital Erlangen Friedrich‐Alexander University Erlangen‐Nürnberg Erlangen Germany; ^7^ Center for Molecular Medicine Cologne (CMMC), Medical Faculty University of Cologne Cologne Germany; ^8^ Cologne Excellence Cluster on Cellular Stress Responses in Aging‐Associated Diseases (CECAD) University of Cologne Cologne Germany; ^9^ Institute of Human Genetics Friedrich‐Alexander University Erlangen‐Nürnberg Erlangen Germany; ^10^ MVZ Dr. Eberhard & Partner Dortmund Dortmund Germany; ^11^ MIRA Vision Microscopy GmbH Göppingen Germany; ^12^ Reference Center for Neuromuscular Disorders Pitié‐Salpêtrière Hospital, AP‐HP Paris France; ^13^ University Hospital of Pediatrics and Adolescent Medicine, St. Josef‐Hospital Ruhr‐University Bochum Bochum Germany

**Keywords:** intermediate filament desmin, myofibrillar myopathy, protein aggregation, proteomicsdesminopathy, secondary mitochondriopathy, transcriptomics

## Abstract

**Background:**

Mutations in the desmin gene cause skeletal myopathies and cardiomyopathies. The objective of this study was to elucidate the molecular pathology induced by the expression of R405W mutant desmin in murine skeletal muscle.

**Methods:**

A comprehensive characterization of the skeletal muscle pathology in hetero‐ and homozygous R405W desmin knock‐in mice was performed. This included grip strength, blood acylcarnitine and amino acid, histological, ultrastructural, immunofluorescence, immunoblot, ribosomal stalling, RNA sequencing and proteomic analyses.

**Results:**

Both hetero‐ and homozygous R405W desmin knock‐in mice showed classical myopathological features of a myofibrillar myopathy with desmin‐positive protein aggregation, degenerative changes of the myofibrillar apparatus and mitochondrial alterations. Muscle weakness and increased blood concentrations of acylcarnitines and amino acids were only present in homozygous animals. During its translation, mutant desmin did not induce terminal ribosomal stalling. Analyses of RNA sequencing and proteomic data from soleus muscle of 3‐month‐old mice depicted 59 up‐ and 3 down‐regulated mRNAs and 101 up‐ and 18 down‐regulated proteins that were shared between the heterozygous and homozygous genotypes in the respective omics datasets compared to the wild‐type genotype. Combined analysis of the omics data demonstrated 187 significantly dysregulated candidates distributed across four groups of regulation. A down‐regulation on the mRNA and protein levels was observed for a multitude of mitochondrial proteins including essential proton gradient‐dependent carriers. Up‐regulation on both omics levels was present for the transcription factor Mlf1, which is a binding partner of protein quality control related Dnajb6. Down‐regulated on mRNA but up‐regulated on the protein level was the sarcomeric lesion marker Xirp2 (xin actin‐binding repeat‐containing protein 2), whereas Ces2c (acylcarnitine hydrolase) was regulated in the opposite way.

**Conclusions:**

The present study demonstrates that the expression of mutant desmin results in a myofibrillar myopathy in hetero‐ and homozygous R405W desmin knock‐in mice. Combined morphological, transcriptomic and proteomic analyses helped decipher the complex pattern of early pathological changes induced by the expression of mutant desmin. Our findings highlight the importance of major mitochondrial alterations, including essential proton gradient‐dependent carriers as well as Dnajb6‐related protein quality control and Xin‐related myofibrillar damage, in the molecular pathogenesis of desminopathies.

## 1 Introduction

Desmin, a muscle‐specific member of the large family of intermediate filament proteins, is a key component of the extrasarcomeric cytoskeleton in muscle cells [S1]. This three‐dimensional filamentous cytoskeletal structure organizes the proper alignment of neighbouring myofibrils at the level of Z‐discs [1], interlinks and attaches the myofibrillar apparatus to costameres in skeletal muscle [2], [S2, S3], to intercalated discs in cardiac cells [S4, S5], and to myotendinous junctions [3] and neuromuscular junctions [4] as well as mitochondria [5], [S6, S7] and nuclei [6, 7], [S8] in striated muscle cells. Beyond these roles in the spatial organization of myofibrils and the positioning of cell organelles, desmin has been reported to exert multiple roles in mechanosensing and stress tolerance [8], [S9], adhesion and migration [9] and cell signalling [10]. The essential role of desmin in human skeletal muscle is highlighted by the observation that mutations in the human desmin gene (*DES*) cause autosomal‐dominant, autosomal‐recessive and sporadic myopathies and cardiomyopathies [1, 11]. In the last three decades more than 100 disease‐causing human *DES* mutations have been described [1], [S10]. The term ‘desminopathies’ comprises all *DES* mutation‐related disorders irrespective of their form of inheritance, mutation type and their consequences on desmin protein expression. Autosomal‐dominant forms, by far the most frequent form of human desminopathies, typically display desmin‐positive protein aggregation pathology and myofibrillar disarray in striated muscle tissue [1]. In the very rare and clinically more severe autosomal‐recessive cases due to homozygous or compound heterozygous *DES* mutations, the issue of desmin and desmin protein aggregation is more complex. In one subform, the desmin protein expression is completely abolished and there is no protein aggregation pathology [12], [S11–S13], whereas in a second and third subform reduced protein levels of solely mutant desmin are seen in conjunction with [13, 14, 15], [S14–S21] or without [16] concomitant signs of protein aggregation. The desminopathies with protein aggregation in striated muscle tissue belong to the clinically and genetically heterogeneous group of myofibrillar myopathies [S22], which is a numerically significant subgroup of human protein aggregation myopathies [S23]. The precise mechanisms leading from the genetic defect to the histopathological lesions observed are poorly understood and, to date, no specific treatment is available for desminopathies and all other forms of myofibrillar myopathies [1].

We previously have reported on the pathogenic effects of the human R406W desmin missense mutation in a young male patient with progressive restrictive cardiomyopathy, cardiac conduction defects and arrhythmias necessitating heart transplantation as well as on the generation and characterization of hetero‐ and homozygous R405W desmin knock‐in mice, which harbour the ortholog of the human R406W desmin mutation [17]. In addition to previously reported patients harbouring this particular missense mutation, who all showed an early disease onset and severe cardiac disease [13, 18, 19, 20], [S24, S25], our work in humans and mice delineated a pronounced cardiotoxic effect of R406W/R405W desmin based on a compromised attachment of structurally altered desmin filaments to intercalated discs [17]. To further decipher the pathology and molecular mechanisms inflicted by mutant desmin, we report here on the comprehensive characterization of the skeletal muscle pathology in hetero‐ and homozygous R405W desmin knock‐in mice by means of grip strength, histological, ultrastructural, immunofluorescence, immunoblot, ribosomal stalling, RNA sequencing and proteomic analyses.

## 2 Materials and Methods

For complete and detailed information on the R405W desmin knock‐in mouse model C57BL/6 N‐*Des* ^tm1.1Allb^ (http://www.informatics.jax.org/allele/MGI:6382571; [17]) and the handling and approved investigation of the mice, measurement of muscle strength, histological evaluation and quantitation of histological parameters, immunoblotting and antibodies, mass spectrometric analysis of acylcarnitine and amino acid levels, transmission electron microscopy, immunofluorescence microscopy and antibodies, mass spectrometry‐based proteomics (PXD042319), RNA sequencing‐based transcriptomics (PRJNA1137105), correlation of proteomic and transcriptomic data, quantitative real‐time PCR, terminal ribosome stalling analysis and image processing, graph generation and figure assembly, please see the ‘**Expanded Materials and Methods**.’ Statistical analyses were performed as indicated in the respective Methods sections and Figure legends. While working on this manuscript, some of the authors found themselves in a similar unfortunate situation to that described in [S26], but discovered that leisure was an effective means of self‐treatment.

## 3 Results

### 3.1 Muscle Weakness, Myopathic Changes and Increased Blood Concentrations of Acylcarnitines and Amino Acids in Homozygous R405W Desmin Knock‐In Mice

**FIGURE 1:**
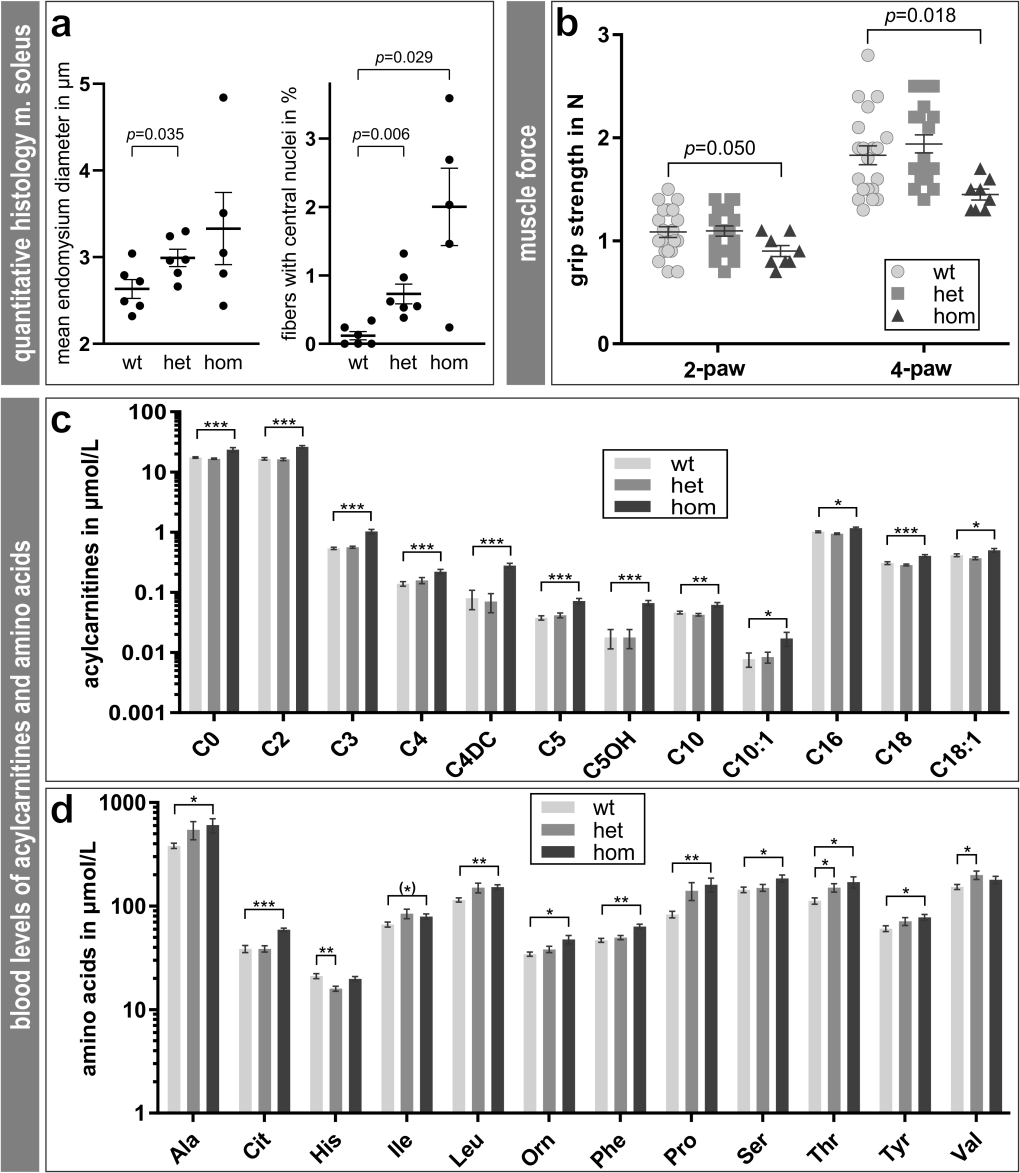
Clinical, histological and biochemical characterization of the skeletal muscle pathology in R405W desmin knock‐in mice. (**a**) Quantitative analysis of the soleus muscle in whole slide images of haematoxylin and eosin–stained muscle sections from 3‐month‐old mice showed a significant increase in endomysium thickness and in the number of fibres with centralized nuclei. See Materials and Methods for details of this analysis. (**b**) Analysis of two‐ and four‐paw grip strengths in cohorts of both sexes of wild‐type (*n* = 20, out of which 14 mice were 3‐ to 4‐month‐old and 6 mice 15‐months of age), heterozygous (*n* = 20, out of which 14 mice were 3‐ to 4‐month‐old and 6 mice 15 months of age) and homozygous (*n* = 8 animals of 3–4 months of age) mice showed significantly decreased force values in the homozygous genotype compared to the wild‐type (Student's *t* test subsequent to two‐way ANOVA). Scatter dot plot with standard error of the mean (SEM). (**c**) Acylcarnitine concentrations determined by mass spectrometry from dried whole blood sample cards derived from heterozygous (*n* = 19) and homozygous (*n* = 14) knock‐in mice and wild‐type littermates (*n* = 18). Multiple acylcarnitines were significantly increased in homozygous animals comprising non‐acetylated carnitine (C0), acetyl‐ (C2), propionyl‐ (C3), butyryl‐ (C4), methyl‐malonyl‐ (C4DC), isovaleryl‐ (C5), 3OH‐isovaleryl‐ (C5OH), decanoyl‐ (C10), cis4‐decanoyl‐ (C10:1), palmitoyl‐ (C16), octodecanoyl‐ (C18) and oleoyl‐ (C18:1) carnitines. (**d**) Amino acid concentrations determined by mass spectrometry from dried whole blood sample cards derived from heterozygous (*n* = 6) and homozygous (*n* = 5) knock‐in mice and wild‐type littermates (*n* = 6). Concentrations of multiple amino acids were also significantly changed, in most cases showing an increase in homozygous mice. (c, d) Bar charts show mean values ± SEM; ^(^*^)^ *p* = 0.055, * *p* < 0.05, ** *p* < 0.01, *** *p* < 0.001; Student's *t* test subsequent to two‐way ANOVA.

To study the effects of R405W mutant desmin expression on skeletal muscle tissue in heterozygous and homozygous knock‐in mice, we first analyzed haematoxylin and eosin–stained transverse sections of the soleus muscles. Myopathological evaluation did not show clear evidence of skeletal muscle myopathy, except for centralisation of myonuclei in 3‐ and 15‐month‐old heterozygous and 3‐month‐old homozygous mice and occasional atrophic fibres in the homozygous samples (Figure S1a–f). Note that old homozygous mice could not be analyzed because they do not survive more than approximately 3 months as described previously [17]. In addition, we subjected whole slide images of the haematoxylin and eosin–stained soleus muscle sections to an automated deep learning‐based image analysis software tool based on photo‐realistic synthetic data in muscle histopathology [21]. This software was used to quantitate diameter, area and number of centralized nuclei of soleus muscle fibres and the endomysium diameter. While this analysis showed no significant changes of fibre diameter and area, increases were detected in endomysium thickness in both genotypes (mean ± SEM; WT 2.6 ± 0.1 μm, HET 3.0 ± 0.1 μm, HOM 3.3 ± 0.4 μm; *n*_WT_ = 6, *n*_HET_ = 6, *n*_HOM_ = 5; *p*_WTvsHET_ = 0.035) and in the fraction of fibres with centralized nuclei (mean ± SEM; WT 0.12 ± 0.06%, HET 0.73 ± 0.14%, HOM 2.00 ± 0.57%; *n*_WT_ = 6, *n*_HET_ = 6, *n*_HOM_ = 5; *p*_WTvsHET_ = 0.006, *p*_WTvsHOM_ = 0.029) (Figure 1a). Additional collagen I immunostaining showed increased staining intensity and broadening of the endomysium in the homozygous sample. This confirms that the slightly thickened endomysium is due to interstitial fibrosis (Figure S1g–i). Myosin heavy chain slow (MHCs) and fast (MHCf) staining showed no significant differences in the fractions of type 1 and type 2 fibres among the three genotypes (Figure S1j–l, and data not shown). Myosin heavy chain developmental (MHCd) and neonatal (MHCn) positive fibres were not detected in any sample (data not shown). We further assessed muscle force production by fore‐limb and four‐paw grip strength measurements, which were performed blinded to the genotype of the mice. This analysis gave a hint of significantly decreased force values in homozygous animals (Figure 1b). The maximal force in two‐paw measurements was reduced by 20% in 3‐month‐old homozygous mice (0.90 ± 0.05 N) compared to wild‐type (1.09 ± 0.05 N) or to heterozygous (1.10 ± 0.05 N) animals. In four‐paw grip strength measurements, an even lower force became apparent in the homozygous mice (1.44 ± 0.05 N) compared to the wild‐type (1.83 ± 0.09 N) or to heterozygous (1.94 ± 0.09 N) animals. Although the results for homozygous animals were statistically significant, we cannot rule out a bias due to reduced motivation in these animals (see the ‘**Expanded Materials and Methods**’ section for details). Mass spectrometry analysis of blood samples showed significantly elevated concentrations of several acylcarnitines (Figure 1c) and amino acids (Figure 1d), indicating altered muscle metabolism in homozygous mice; see the ‘**Expanded Results**.’

### 3.2 Myofibrillar and Protein Aggregate Pathology, Vacuolar Structures and Mitochondrial Abnormalities: Ultrastructural Features in Hetero‐ and Homozygous R405W Desmin Knock‐In Mice

**FIGURE 2:**
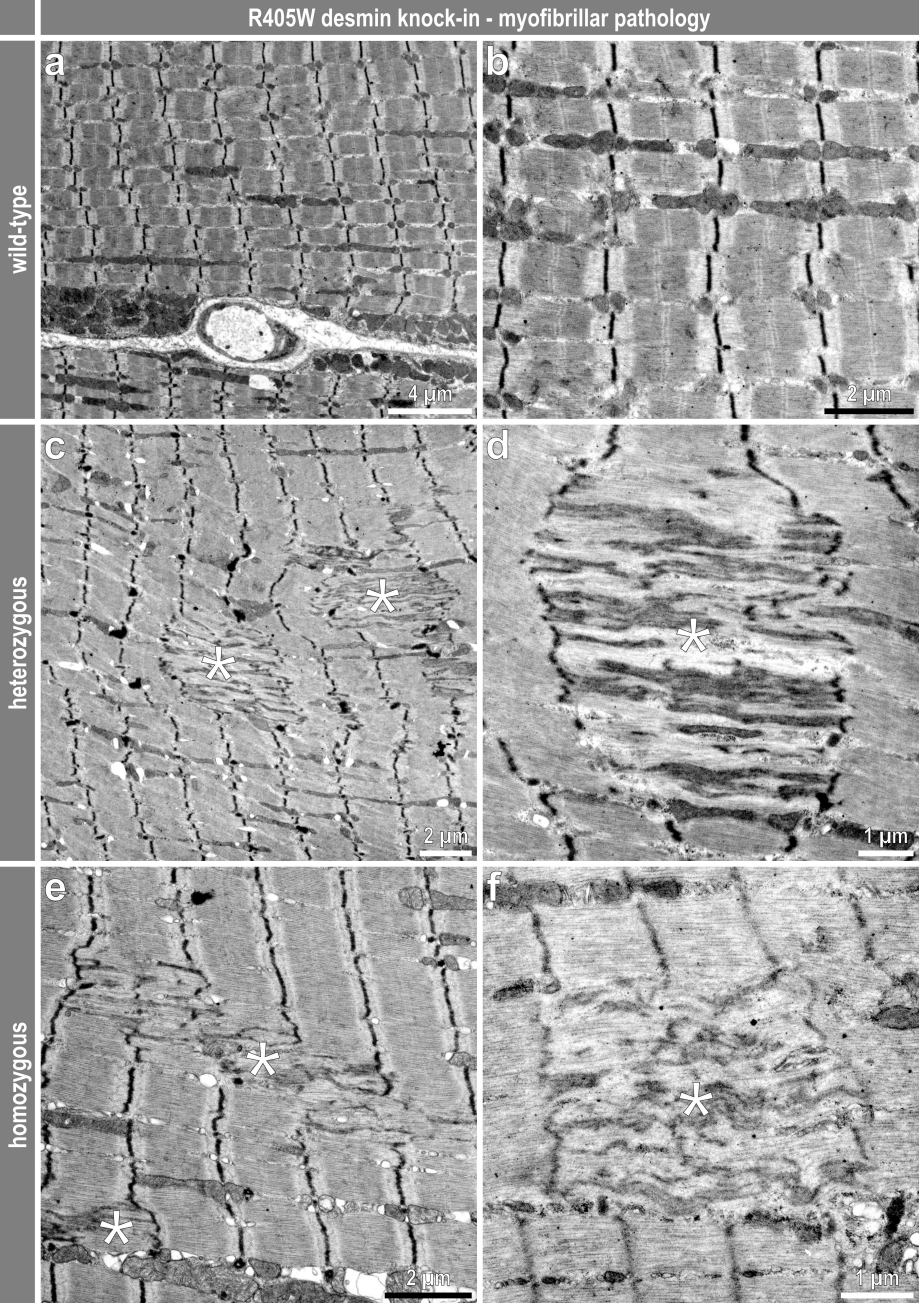
Myofibrillar pathology in skeletal muscle of R405W desmin knock‐in mice. (**a, b**) Electron micrographs of normal myofibrillar and sarcomeric architecture in soleus muscle of wild‐type mice. (**c–f**) Sarcomeric lesions (asterisks) affecting multiple neighbouring myofibrils were frequently observed in soleus muscle of hetero‐ and homozygous R405W desmin knock‐in mice. These lesions spanned from Z‐band to Z‐band and typically affected between one to five sarcomeres in longitudinal orientation.

Though force measurements in living mice and haematoxylin and eosin–stained sections from soleus muscles showed no overt pathology in heterozygous animals, their ultrastructural examination disclosed all the typical signs of a myofibrillar myopathy comprising sarcomeric lesions with Z‐band streaming (Figure 2a–d), subsarcolemmal and intermyofibrillar protein aggregates (Figure S2a, b, d) as well as cytoplasmic bodies (Figure S2c). Additional typical features were the presence of multiple large vacuolar structures (Figures S2b, 3a, b) and accumulation of both normally and atypically shaped mitochondria (Figures S2a–c, 3a,b). All these myofibrillar myopathy typical features were also present in homozygous R405W desmin knock‐in mice, however, at a much more pronounced level, including the myofibrillar (Figure 2e, f) and protein aggregation (Figure S2e, g) pathology, and abnormally shaped and enlarged mitochondria (Figure S2e–h, 3c,d ). In contrast to the heterozygous genotype, the ultrastructural analysis in the homozygous genotype also revealed the presence of protein aggregates composed of electron dense material (Figure S2f, h) and filamentous material (Figure S2g).

**FIGURE 3:**
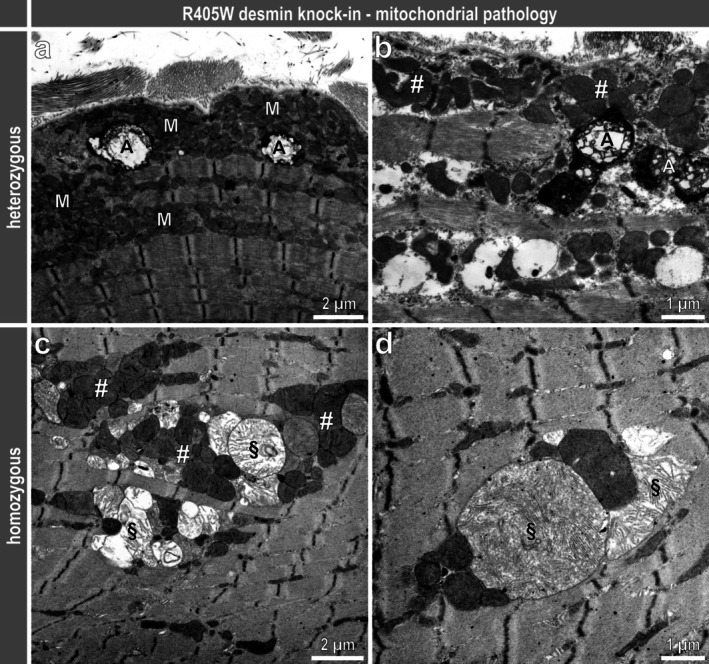
Mitochondrial pathology in skeletal muscle of heterozygous and homozygous R405W desmin knock‐in mice. (**a**) Subsarcolemmal and intermyofibrillar accumulation of mitochondria (M) and large vacuolar structures that in part contain electron‐dense material (A). (**b**) Highly pleomorphic mitochondria (#) and large vacuolar structures (A). (**c**) Intermyofibrillar accumulation of pleomorphic mitochondria (#). Note the enlargement of mitochondria and the dissolution of mitochondrial cristae (§). (**d**) Intermyofibrillar giant mitochondria (higher magnification revealed the mitochondrial double‐membrane) with widened and abnormal cristae (§).

### 3.3 Desmin Immunofluorescence Stains Depict Dot‐Like Protein Aggregates in Skeletal Muscle From Hetero‐ and Homozygous R405W Desmin Knock‐In Mice

**FIGURE 4:**
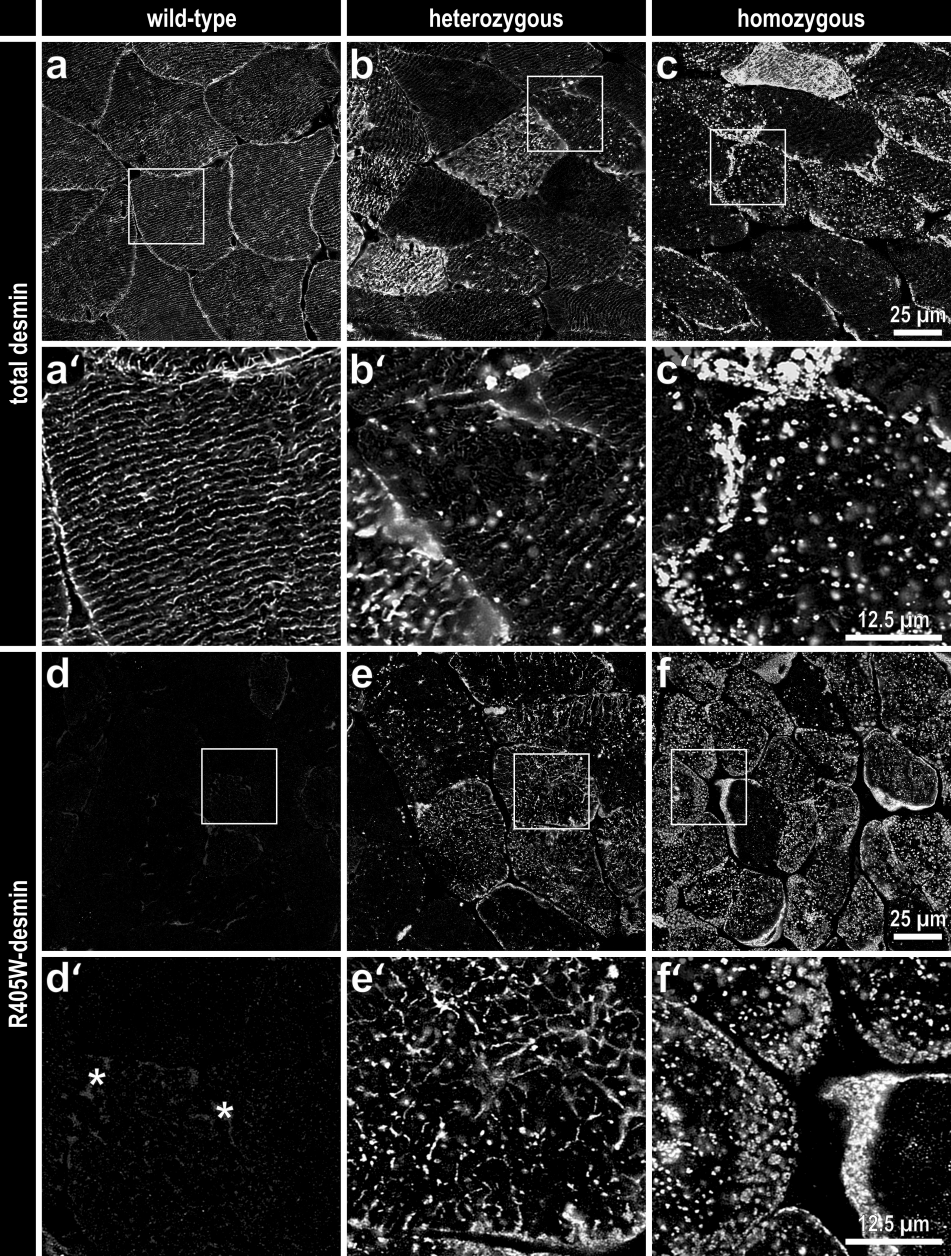
Desmin‐positive protein aggregation in skeletal muscle of heterozygous and homozygous R405W desmin knock‐in mice. (**a–c**) Desmin immunostaining using a desmin antibody detecting both wild‐type and mutant desmin (total desmin) of transverse sections of soleus muscle from heterozygous and homozygous R405W desmin knock‐in mice and wild‐type littermates. Note the attenuation of the normal desmin cross‐striation pattern as well as the presence of small roundish desmin‐positive aggregates in heterozygous and more pronounced in homozygous muscle. (**a’–c’**) Areas with a higher magnification. (**d–f**) Analogous immunostaining using a 405 W‐specific desmin antibody, which visualizes small R405W desmin aggregates in the soleus muscle of heterozygous and homozygous mice. (**d’–f’**) Areas with a higher magnification. Asterisks, low‐intensity autofluorescence in the subsarcolemmal region of the wild‐type soleus muscle. Images were deconvolved using Huygens Essential (Scientific Volume Imaging, Hilversum, the Netherlands).

In line with the detection of protein aggregates at the ultrastructural level, desmin immunofluorescence stains demonstrated the presence of multiple dot‐like desmin‐positive protein aggregates in transverse sections of soleus muscle derived from both hetero‐ and homozygous R405W desmin knock‐in mice (Figure 4). In tissue from heterozygous mice, the small desmin‐positive aggregates were less abundant than in the homozygous genotype, and the desmin cross‐striated staining pattern was still present but attenuated in multiple fibres, whereas it was abolished in virtually all fibres from homozygous animals (Figure 4a–c’). These findings were further highlighted by using a 405 W–specific desmin antibody (Figure 4d–f’).

### 3.4 Expression of Mutant Desmin Resulted in the Detection of Only 62 Dysregulated mRNAs Shared Between Hetero‐ and Homozygous R405W Desminopathy Mice

**FIGURE 5:**
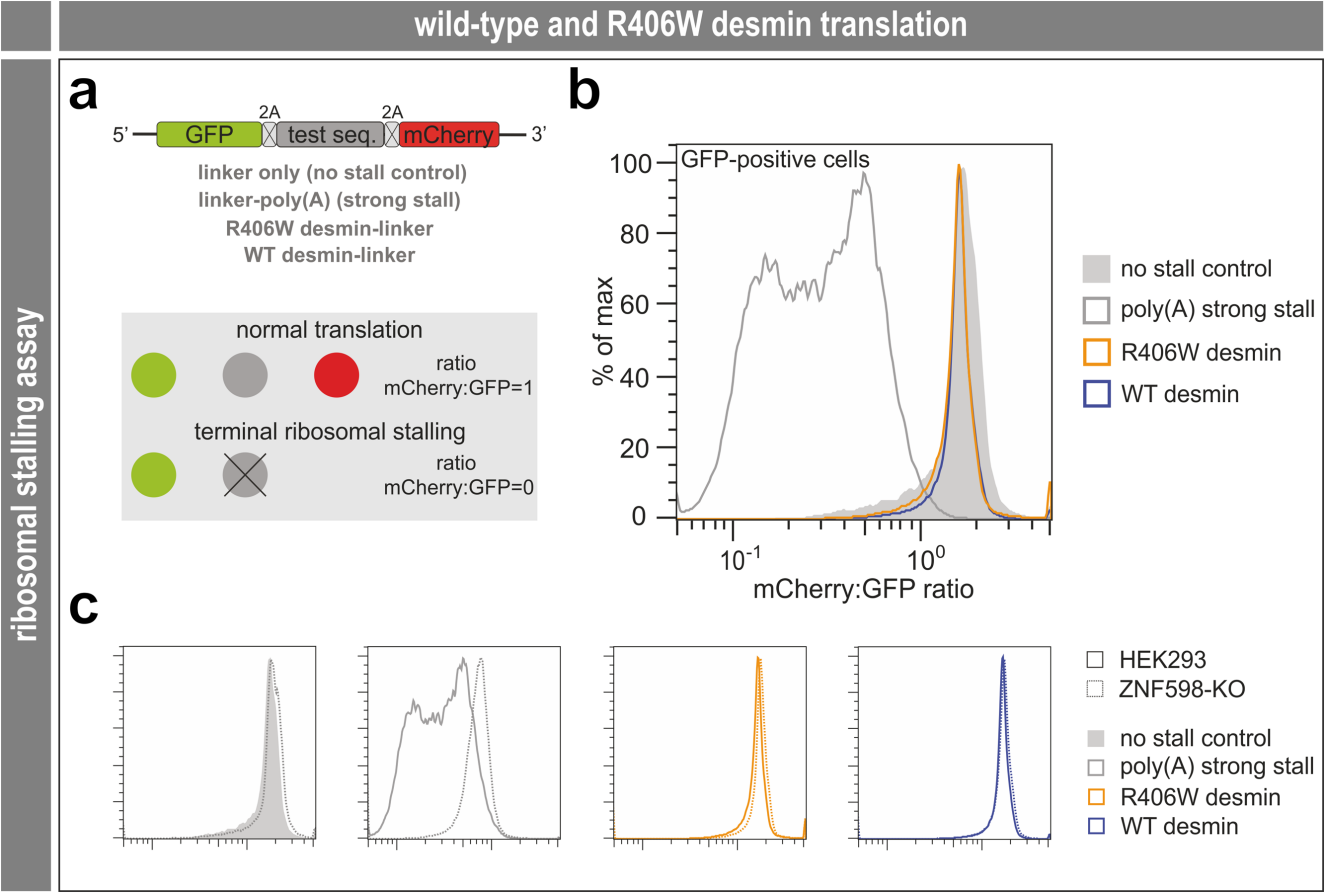
Analysis of R406W mutant desmin translation. (**a**) Dual fluorescence reporters for measuring terminal ribosomal stalling. The test sequence is placed between two fluorescent proteins, GFP and mCherry, separated by viral 2A peptides that induce ribosomes to skip the formation of one peptide bond without interrupting translation [S42]. Problems in ribosome elongation during the synthesis of the test sequence lead to abortion of translation, preventing the synthesis of mCherry. This results in decreased mCherry to GFP fluorescence ratios in flow cytometry analysis. As reference, we also measured two previously published reporters, an unstructured linker sequence (no stall control) and a linker containing a poly(A) sequence (20xAAA^Lys^, a strong inducer of ribosome stalling) [S43]. (**b**) Analysis of human wild‐type (WT) and R406W desmin translation in HEK293 cells. Histogram depicts the mCherry/GFP fluorescence ratios of GFP positive cells after 48 h of transient transfection. Note that neither wild‐type nor mutant desmin caused ribosomal stalling. (**c**) Comparison of terminal ribosomal stalling in WT (as in b) and in ZNF598 knockout cells, where rescue of stalled ribosomes is abrogated.

**FIGURE 6:**
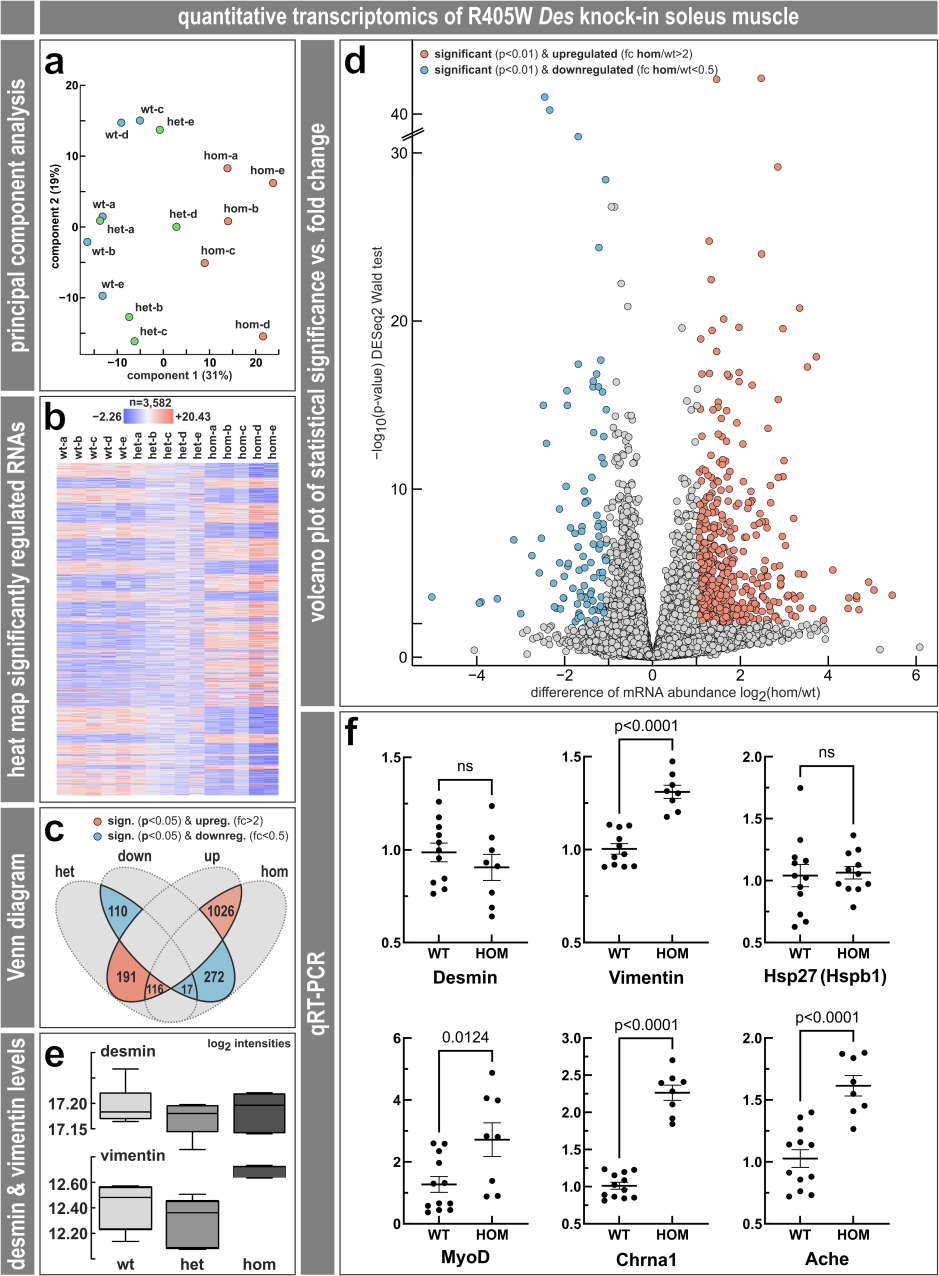
RNAseq data analysis of soleus muscle tissue derived from R405W desmin knock‐in mice. (**a**) RNAseq principal component analysis. Principal components 1 and 2 are plotted for all five mice of each genotype (wild‐type in blue, heterozygous in green, homozygous in orange). PC1 accounts for 31% of the variance and PC2 accounts for 19% of the variance in these data and separated homozygous R405W desmin knock‐in muscle from the wild‐type and heterozygous conditions. (**b**) Heatmap of the hierarchical clustering of all significantly regulated RNAs in soleus muscle (*n* = 3582) indicating decreased levels in blue and increased levels in orange. (**c**) Venn diagram comparison [S44] of the number of significantly (*p* < 0.05) and markedly (fc > 2, fc < 0.5) up‐ and down‐regulated RNAs detected by DESeq2 RNAseq data analysis. (**d**) Volcano plot of significantly differentially expressed mRNAs in homozygous muscle. Criteria of *p* value < 0.01 and fold change > 2 or < 0.5: orange, up‐regulated; blue, down‐regulated. (**e**) Box plot illustrations of expression of *Des* and *Vim* in the three genotypes. Y‐axis, mean DESeq2 values. (**f**) qRT‐PCR analysis of *Des*, *Vim*, *Hsp27, MyoD*, *Chrna1* and *Ache* expression in R405W homozygous mice compared to wild‐type. Y‐axis, normalized expression ratio (2^−ΔΔCq^).

The above findings showed that the mono‐ and bi‐allelic expression of the R405W mutant desmin led to a myofibrillar myopathy with desmin‐positive protein aggregates and degenerative changes of the myofibrillar apparatus. To study the molecular pathophysiology of this protein aggregate myopathy in more detail, we next analyzed the ribosomal translation of R405W/R406W desmin and the global, R405W desmin‐induced changes on the RNA and protein levels by transcriptomics and proteomics. Measurement of the incidence of terminal ribosomal stalling (Figure 5a) showed that the rates of full‐length synthesis are the same for wild‐type and R406W mutant desmin (Figure 5b) and that desmin translation is unaffected by knocking‐out the ribosome collision sensor ZNF598 (Figure 5c); see the ‘**Expanded Results**.’ RNA sequencing (RNAseq) using soleus muscle derived from hetero‐ and homozygous R405W desminopathy mice and wild‐type littermates let to a detection of a total of 29 616 different RNA species in all three genotypes, of which 17 538 were protein‐coding (Table S2); see the ‘**Expanded Results**’ for further details. Principal component analysis of the data derived from the five mice per genotype showed two different clusters, one with the homozygous R405W soleus samples separated from the second containing the wild‐type and heterozygous genotypes (Figure 6a). Normalized counts of the top 3582 significantly (based on the corrected *p* values derived from the homozygote versus wild‐type comparison) regulated RNAs were plotted in heat‐map form indicating a high transcriptional heterogeneity between the homozygous and wild‐type samples (Figure 6b). Within the limits of a significant (*p* < 0.05) and > 2‐fold regulation, 1142 (1026 + 116) RNA species were up‐ and 289 (272 + 17) down‐regulated in homozygous and 307 (191 + 116) up‐ and 127 (110 + 17) down‐regulated in heterozygous soleus muscle compared to the wild‐type. However, only 116 up‐ and 17 down‐regulated RNAs were shared between heterozygous and homozygous muscles (Figure 6c). A volcano plot of differentially expressed, protein‐coding RNAs (*p* < 0.01, >2‐fold regulation) in homozygous and wild‐type soleus muscle illustrated 627 up‐regulated mRNAs and a number of 104 down‐regulated mRNA species (Figure 6d, Table S4). Out of these 731 mRNAs, only 59 and 3 mRNAs showed an up‐ and down‐regulation, respectively, shared between the heterozygous and homozygous genotypes (Table S4, 1st sheet, in bold orange and blue; Table S4, 2nd sheet). These ‘double‐hits’ comprised diverse mRNAs related to various cellular functions, for example, extracellular matrix (Icam4, Mmp3, Mmp13, Fsbp), reactive oxygen species metabolism (Mpv17l), smooth muscle differentiation (Olfm2), mitochondria (Cyb5r2, Smim5) and Ca^2+^/sarcoplasmic reticulum (Ryr3). Finally, we analyzed and validated the mRNA levels of a few genes of interest by qRT‐PCR. In line with the RNAseq results for desmin and vimentin (Figure 6e), desmin levels by qRT‐PCR were similar in wild‐type and homozygous R405W desmin knock‐in soleus muscle (fold change desmin, qRT‐PCR 0.9, RNAseq 1.0), while vimentin displayed a significant, moderate increase (fold change vimentin, qRT‐PCR 1.3 with *p* < 0.0001, RNAseq 1.4 with *p* < 0.00624) (Figure 6f). Further candidates also showed a similar up‐regulation in qRT‐PCR and RNAseq (fold change MyoD, qRT‐PCR 2.1 with *p* < 0.0124, RNAseq 3.0 with *p* < 0,0039; Chrna1, qRT‐PCR 2.2 with *p* < 0.0001, RNAseq 1.6 with *p* < 0.0001; Ache, qRT‐PCR 1.6 with *p* < 0.0001, RNAseq 1.5 with *p* < 0.0001) (Figure 6f, Table S2), while Hsp27 was not significantly regulated using either of the methods (fold change Hsp27, qRT‐PCR 1.0, RNAseq 1.1).

### 3.5 Expression of Mutant Desmin Led to the Detection of 119 Dysregulated Proteins Shared by Hetero‐ and Homozygous R405W Desminopathy Mice

**FIGURE 7:**
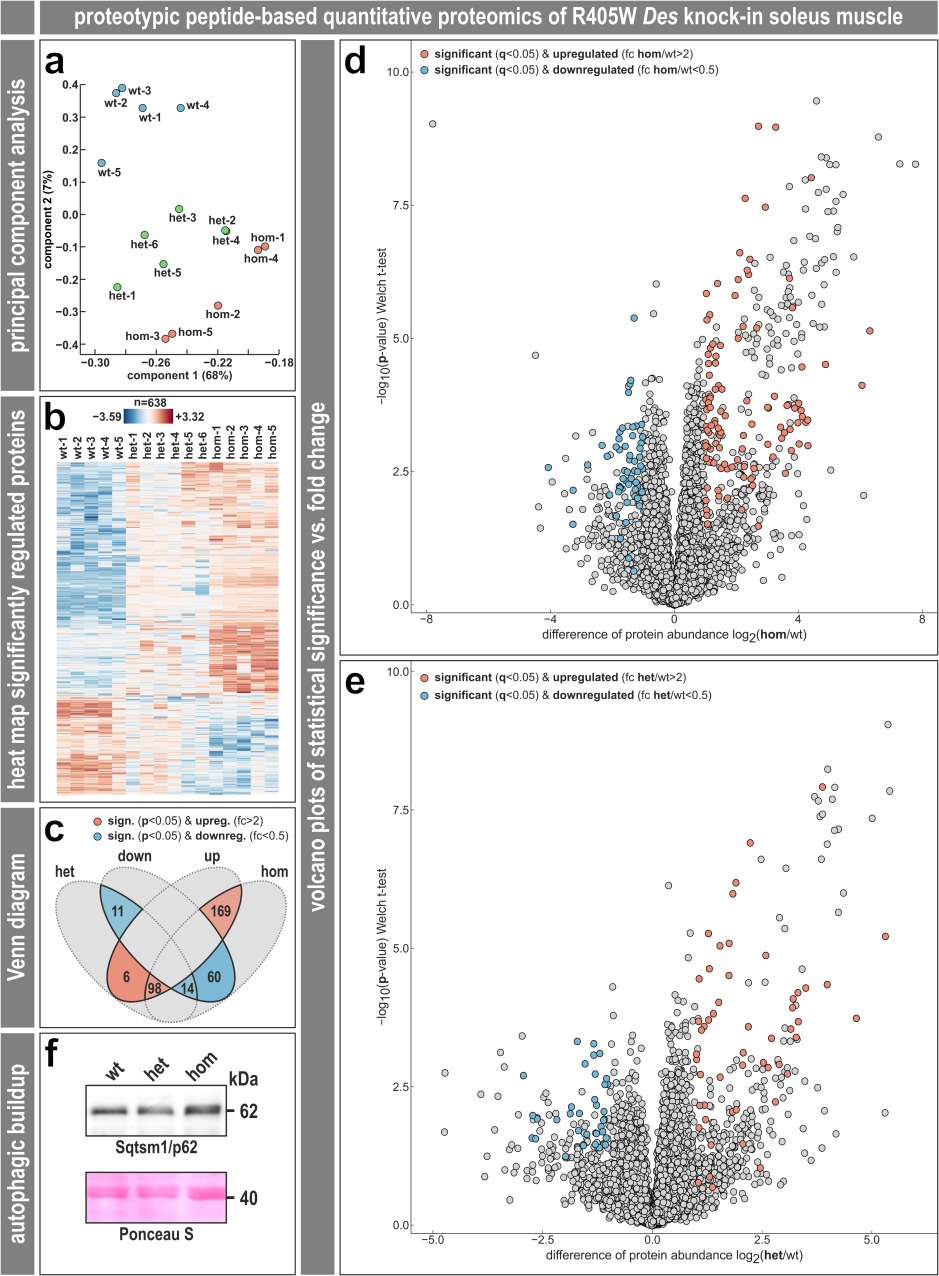
Proteotypic peptide‐based quantitative proteomic analysis of soleus muscle tissue derived from R405W desmin knock‐in mice. (**a**) Principal component analysis (PCA), which determined main differences in the global protein expression pattern, showed a clear separation of the three genotypes (wild‐type 1–5 in blue, heterozygous 1–6 in green, homozygous 1–5 in orange). (**b**) Heat map of all significantly regulated proteins (*n* = 638) indicating decreased protein levels in blue and increased levels in orange. (**c**) Venn diagram summarizing the numbers of significantly (*p* < 0.05) and markedly (fc > 2, fc < 0.5) up‐ and down‐regulated proteins in the comparisons between heterozygous versus wild‐type and homozygous versus wild‐type. Note that the number of upregulated proteins in both heterozygous and homozygous soleus muscle (98) clearly exceeds the number of downregulated proteins (14). (**d**, **e**) Volcano plots comparing protein levels of homozygous versus wild‐type (d) and heterozygous versus wild‐type genotypes (e). X‐axis, log_2_‐transformed mean difference of protein abundance (fold change); y‐axis, −log_10_ transformed *p* value. Proteins with a statistical significance of ANOVA *q* < 0.05 (adjusted *p* value) and a regulation of 2‐fold (fc > 2 in orange, fc < 0.5 in blue) are shown in coloured dots. Note that some dots that appear to be significant based on their *p* value dependent position in the plot are not coloured due to their *q* value. (**f**) Sqstm1/p62 immunoblotting of lysates of gastrocnemius muscles from 3‐month‐old mice. The Sqstm1/p62 protein levels were significantly increased in muscles from homozygous mice, as shown by an exemplary immunoblot (see the Results section for more information). Ponceau S stained immunoblot membrane as loading control.

A global proteomic analysis also using soleus muscle tissue derived from hetero‐ and homozygous R405W desminopathy mice and wild‐type littermates resulted in the detection of a total of 4517 different proteins identified by proteotypic peptides (Table S5, 1^st^ sheet); see the ‘**Expanded Results**’ for further details. In contrast to the RNA level, the principal component analysis of the proteomic data determined main differences in the global protein expression pattern with a clear separation of all three genotypes (Figure 7a). Mean intensity values of the 638 significantly regulated proteins (based on ANOVA *q* < 0.05 across the three genotypes [Table S5, 4th sheet]) were plotted in heat‐map form, which clearly separated the three genotypes (Figure 7b). In the Venn diagram of significantly (*p* < 0.05) and > 2‐fold regulated proteins, 267 (169 + 98) proteins were up‐ and 74 (60 + 14) down‐regulated in homozygous and 104 (98 + 6) up‐ and 25 (14 + 11) down‐regulated in heterozygous soleus muscle compared to the wild‐type, and the number of up‐regulated proteins in both heterozygous and homozygous soleus muscle (*n* = 98) clearly exceeded the number of down‐regulated proteins (*n* = 14) (Figure 7c). Volcano plots of differentially expressed proteins (ANOVA *q* < 0.05, >2‐fold regulation) illustrated 60 and 127 up‐ and 35 and 65 down‐regulated proteins in the hetero‐ and homozygous conditions, respectively, compared to wild‐type (Figure 7d, e; Table S5, 5th and 6th sheet). Out of the significantly regulated proteins (*q* < 0.05 for het vs. wt and for hom vs. wt single comparisons, >2‐fold regulation), 46 were up‐regulated and 16 were down‐regulated ‘double‐hits’ shared between the heterozygous and homozygous genotypes. Moreover, also with significance (*q* < 0.05) but without a fold change (see the ‘**Expanded Results**’), 55 additional proteins were expressed in both hetero‐ and homozygous conditions but not in the wild‐type, and a further two proteins were expressed in the wild‐type but not in the hetero‐ or homozygous conditions (Table S5, the 119 entries in bold, and Table S5, 7th sheet). The latter two proteins not detected in both desminopathy genotypes were Tep1 (telomerase protein component 1) and Mrpl33 (mitochondrial 39S ribosomal protein L33, large ribosomal subunit protein bL33m). The 16 double‐down‐regulated proteins comprised, for example, the intermediate filament proteins Krt19 and Syne2, the cytoskeletal proteins Macf1, Myh10 and Obscn, the mitochondrial proteins Slc25a4 (ADP/ATP translocase 1) and Marc2, and Slc27a1 (long‐chain fatty acid transport protein 1) (Table S5, 7^th^ sheet, lower part). The 101 double‐upregulated/expressed proteins (46 + 55) (Table S5, 7th sheet, upper part) comprised, for example, the intermediate filament protein Sync, multiple proteins of the quality control such as Atg101, Bag3, Dnajb6, Gan, Klhl21, Klhl38, Ulk1, Usp2, including several E3 ubiquitin‐protein processing proteins (Maea, March7, Rmnd5a, Sh3rf2, Trim35, Uchl1, Ufc1), Nup133 (nuclear pore complex protein), the mitochondrial proteins Atp5mc1 and Cox7b, Casq1 (calsequestrin‐1) and the transcription factors Mlf1 and Mlf2 (myeloid leukaemia factors 1 and 2).

As the electron micrographs showed multiple large vacuolar structures in heterozygous and more pronounced in homozygous soleus muscle, we complemented the proteomic analysis with quantitative immunoblotting of p62/sequestosome‐1, which is a main marker of the autophagic pathway. Because the soleus muscles were used up for transcriptomics and proteomics, we used total protein extracts from gastrocnemius muscles of the same mice for immunoblotting. We detected a significant increase of p62/sequestosome‐1 (1.6‐fold, hom vs. wt, *p* = 0.005; samples from 10 mice of each genotype were analyzed in triplicate by Western blotting) in homozygous R405W desmin knock‐in mice (Figure 7f). This finding is in line with the proteomic analysis, which showed a significant 1.75‐fold up‐regulation in homozygous soleus muscle (Table S5).

### 3.6 Correlation Analysis of Transcriptomic and Proteomic Datasets Led to a Number of 187 Significantly Dysregulated Genes in R405W Desmin Muscles

Exploiting our transcriptomic and proteomic datasets, we next looked for a correlation between the datasets by using both bioinformatic and manual analyses. For the former, the analysis was restricted to the homozygous and wild‐type genotypes. Here, mRNA and corresponding protein list entries were adjusted for mRNA multimapping in the case of 32 proteins. A linear model was used to analyse the correlation between the transcriptomic and proteomic datasets, using only entries generated in this way that were unique and present in both datasets (*n* = 4230), resulting in *R* ^2^_adj_ = 0.041 with a statistical significance of *p* = 0.003. Dot plot visualization of the combined transcriptomic and proteomic datasets with a significance level of *q* < 0.05 set for the mRNA and corresponding protein list entries resulted in four different groups of regulation (Figure S3). One group (orange dots) contained mRNAs and corresponding proteins that were both up‐regulated (*n* = 32), including the intermediate filament proteins Sync and Lmnb2, Trim7, the nuclear envelope‐related protein Tmem43 (transmembrane protein 43), the transcription factor Mlf1 (myeloid leukaemia factor 1) and the sarcolemma and extracellular matrix‐associated proteins Itga5 (integrin alpha‐5) and Lum (lumican) (Figure S3, Table S7). A second group (blue dots) represented mRNAs and corresponding proteins that were both down‐regulated (*n* = 90) and consisted of about 50% mitochondrial proteins. This group included, for example, the fatty acid metabolism‐related proteins CD36 (leukocyte differentiation antigen CD36, platelet glycoprotein 4, fatty acid translocase, glycoprotein IIIb), Slc25a20 (mitochondrial carnitine/acylcarnitine carrier protein), Acadsb (short/branched chain specific acyl‐CoA dehydrogenase), Acadm (medium‐chain specific acyl‐CoA dehydrogenase) and Slc27a1 (long‐chain fatty acid transport protein 1), Vdac1 and the mitochondrial proton gradient‐dependent carriers Slc25a3 (mitochondrial phosphate carrier protein), Slc25a4 (ADP/ATP translocase 1) and Slc25a12 (calcium‐binding mitochondrial carrier protein Aralar1, mitochondrial electrogenic aspartate/glutamate antiporter SLC25A12) (Figure S3, Table S7). In addition to the two groups with same‐sense correlations, there were also two groups with opposing correlations. One group (yellow dots) represented genes up‐regulated on the mRNA but down‐regulated on the protein level (*n* = 13), including Krt19 (type I cytoskeletal keratin 19) and Ces2c (acylcarnitine hydrolase) (Figure S3, Table S7). The other group (green dots) represented genes down‐regulated on mRNA but up‐regulated on protein level (*n* = 52), including Xirp2 (xin actin‐binding repeat‐containing protein 2) (Figure S3, Table S7).

The manual analysis of the 4230 entries of the combined transcriptomic and proteomic datasets was based on known desmin‐related protein interactions, myofibrillar myopathy–related proteins, proteins that are enriched in protein aggregates in desminopathies and cellular compartments and processes known to play a role in desminopathies. Candidates of interest were sorted into the following categories, ‘intermediate filament & associated,’ ‘sarcomere,’ ‘heat shock related,’ ‘protein quality control,’ ‘mitochondria,’ ‘oxidative stress,’ ‘mechanical stress,’ ‘neuromuscular junction,’ ‘channels & transporters,’ ‘myogenesis & regeneration,’ ‘cell cycle,’ ‘nuclear envelope,’ ‘adhesion & migration & extracellular matrix,’ ‘inflammation,’ and ‘other candidates of interest’ (Table S8). Direct desmin interaction partners [1, 22], [S27] that were detected (*n* = 17) were indicated with an asterisk (Table S8, column Q; Table S8, 2nd sheet, column Q). Out of the group of ‘intermediate filament and intermediate filament associated proteins,’ which harbours several direct desmin interaction partners, only two candidates, namely, keratin 19 and syncoilin, were significantly regulated in both the heterozygous and homozygous genotypes on the protein level and additionally in the homozygous genotype on the mRNA level. Using the same criteria, cytochrome c oxidase subunit 7B, ATP synthase subunit a, mitochondrial fission regulator 1‐like, succinate dehydrogenase cytochrome b small subunit and ADP/ATP translocase 1 were present in the group of ‘mitochondrial proteins,’ selenoprotein W in the group of ‘oxidative stress,’ and calcium/calmodulin‐dependent protein kinase type II subunit beta and calsequestrin‐1 in the ‘other candidates of interest’ (Table S8). When focusing on the heterozygous genotype, which is comparable to the human disease, we detected not a single candidate that was regulated on both the protein and mRNA levels. Out of the genes whose mutations cause myofibrillar myopathies or protein aggregation myopathies (*n* = 16) [23], [S28] (Table S8, column R; Table S8, 2nd sheet, column R), only two candidates, namely, Dnajb6 and Bag3, were up‐regulated on the protein level in both the heterozygous and homozygous phenotypes. When looking for proteins that were reported to be enriched in fibres containing protein aggregates in skeletal muscle tissue from desminopathy patients (*n* = 19) [24] (Table S8, column S; Table S8, 2nd sheet, column S), Mlf2 (myeloid leukaemia factor 2) was up‐regulated on the protein level in both the heterozygous and homozygous R405W desmin knock‐in mice, and Psmb4, Xirp2 and Dcxr only in the homozygous phenotype.

## 4 Discussion

For discussion of the increased blood concentrations of acylcarnitines and amino acids in homozygous R405W desmin knock‐in mice, which could serve as biomarkers of secondary mitochondrial dysfunction in autosomal‐recessive desminopathies, and the presence of alterations in the UFMylation machinery in hetero‐ and homozygous mice, but the absence of abortive translation during the synthesis of R406W mutant desmin protein, see the ‘**Expanded Discussion.**’

### 4.1 R405W Desmin Causes a Myofibrillar Myopathy in Both Hetero‐ and Homozygous Desmin Knock‐In Mice

Extending our previous work on the cardiotoxic effects of R406W/R405W mutant desmin in humans and mice [17], we report here a comprehensive characterization of the skeletal muscle pathology in hetero‐ and homozygous R405W desmin knock‐in mice. The immunofluorescence and ultrastructural analyses depicted the pathognomic features of myofibrillar myopathies [S22] comprising desmin‐positive protein aggregates, degenerative changes of the myofibrillar apparatus, vacuolar structures and mitochondrial abnormalities. These classical alterations were present in both the heterozygous genotype and the homozygous R405W desmin knock‐in mice. The former are the genetic equivalent of the autosomal‐dominant human R406W desminopathy [1, 13, 17, 18, 19, 20], [S24, S25,] and the latter are a surrogate for the very rare autosomal‐recessive human desminopathies with maintained mutant desmin protein expression [S29]. Notable is the fact that neither hetero‐ nor homozygous animals displayed morphological signs of protein aggregation pathology in standard H&E and Gömöri trichrome stains and that muscle weakness was only present in homozygous R405W desmin knock‐in mice, which reach their age limit at 3–4 months due to a lethal intestinal pseudo‐obstruction [17]. Thus, these findings are analogous to the previously published observations in R349P desmin knock‐in mice [25] and suggest that ultrastructural changes in muscle fibres, including but not limited to protein aggregation, precede and probably underlie the development of detectable loss of muscle force.

### 4.2 Noxious Effects of R405W Desmin on the Intermediate Filaments, the Myofibrillar System and Mitochondria

Previous in vitro assessment of the assembly properties of R406W desmin in equimolar mixtures with wild‐type desmin showed the formation of chimeric filaments with normal morphology and interspersed sections of various irregularities, while the assembly of R406W desmin alone resulted in the formation of thickened filaments, aggregates and fibrillar sheets [17]. The in vitro effects of the R406W mutant desmin were mirrored in vivo by desmin immunostains, which depicted multiple dot‐like desmin‐positive protein aggregates in the skeletal muscle of hetero‐ and homozygous R405W desmin knock‐in mice with greater abundancy in the latter (Figure 4). A cross‐striated desmin staining pattern, although often attenuated, was found only in the skeletal muscle of heterozygous animals (Figure 4b’), suggesting that the formation of chimeric filaments, as seen in the in vitro analysis, still allows the formation of a three‐dimensional but abnormal desmin cytoskeleton. Because the cross‐striated desmin immunolabelling pattern was almost completely absent in the homozygous genotype (Figure 4c’), the structure and function of the desmin filament system appears to be virtually abolished. On the ultrastructural level these subsarcolemmal and intermyofibrillar protein aggregates displayed a complex picture with granulo‐filamentous (Figure S2a), filamentous (Figure S2c, g), granular (Figure S2d) and electron‐dense (Figure S2f, h) appearances and varied in size and number between individual muscle fibres, reminiscent to the granulo‐filamentous inclusions present in human desminopathies [1, 26], [S22]. The presence of filamentous inclusions in homozygous animals (Figure S2g) possibly represent the equivalent of the thickened filament structures formed by pure R406W desmin in vitro [17]. The faulty R406W/R405W mutant desmin assembly process and its impact on the structure and function of the extrasarcomeric cytoskeleton thus provides an explanation for numerous subsequent pathological lesions in desminopathies comprising i) formation of protein aggregates, ii) defects in the proper alignment and anchorage of the myofibrillar apparatus with concomitant mechanical strain‐induced degenerative alterations and iii) defects in the distribution, morphology and function of mitochondria. The fact that these key pathological lesions are also present in skeletal muscle tissue of autosomal‐dominant and ‐recessive human desminopathies [1], [S22] as well as in hetero‐ and homozygous R349P desmin knock‐in mice [6, 25, 27], [S30, S31] underscores the importance of such pathogenetic sequence in desminopathies.

### 4.3 Transcriptomic and Proteomic Datasets Highlight Changes in Mitochondrial Function and Protein Quality Control in 3‐Month‐Old R405W Desminopathy Mice

To assess disease‐relevant changes, we used soleus muscle tissue from three‐month‐old heterozygous and homozygous R405W desmin knock‐in mice and wild‐type littermates to perform RNA sequencing and quantitative mass spectrometry‐based proteomics. In the heterozygous genotype and much more pronounced in the homozygous genotype mRNAs were up‐ and down‐regulated in approximately equal proportions. However, only the homozygous genotype clearly separated in the principal component analysis. On the protein level, dysregulation was also more prominent in the homozygous genotype, but markedly more proteins were up‐ than down‐regulated, and all three genotypes were clearly separated in the principal component analysis.

A very prominent group of dysregulated candidates comprised mitochondrial proteins. Of particular interest was the down‐regulation of the mitochondrial proton gradient‐dependent carriers Slc25a3 (phosphate carrier), Slc25a4 (ADP/ATP translocase 1) and Slc25a12 (calcium‐binding carrier Aralar1, electrogenic aspartate/glutamate antiporter), all of which have been described to be down‐regulated in mitochondria purified from R349P desminopathy myotubes and/or in soleus muscle tissue from R349P desmin knock‐in mice ([28] and supplementary tables therein). Down‐regulation of these proteins provided an explanation for the detection of a reduced proton leak in mitochondria from cultivated R349P desminopathy myotubes by means of high‐resolution respirometry [28]. Reduction of these highly essential carriers affects the normal mitochondrial ATP production [S32] and very likely triggers a damaging cascade comprising electron transport chain overload and associated reactive oxygen species production [29] and mtDNA damage and deletions [30], [S33, S34], ultimately resulting in functionally and structurally compromised mitochondria. Accordingly, our ultrastructural analysis showed enlarged mitochondria with widened cristae in the soleus muscle of R405W (Figure 3) and R349P [27] desminopathy mice. Furthermore, the down‐regulation of Mrpl33 (mitochondrial 39S ribosomal protein L33, large ribosomal subunit protein bL33m) is of interest as it is a component of the mitochondrial ribosome and its knock‐down was reported to cause a decrease in ATP production and an increase in reactive oxygen species [31], [S35]. Notably, a homozygous mutation in *SLC25A3* has been identified as cause of reduced mitochondrial ATP production in muscle, leading to mitochondrial phosphate carrier deficiency with lactic acidosis, hypertrophic cardiomyopathy and muscle hypotonia in humans [32]. Moreover, a homozygous mutation in *SLC25A4* has been found to cause myopathy and cardiomyopathy with multiple mtDNA deletions in skeletal muscle tissue [33].

With regard to the increased blood concentrations of multiple acylcarnitines, the down‐regulation of fatty acid metabolism‐related proteins is noteworthy. In particular, we detected reduced amounts of the fatty acid translocase CD36 (glycoprotein IIIb, leukocyte differentiation antigen CD36), Slc27a1 (long‐chain fatty acid transport protein 1), Ces2c (acylcarnitine hydrolase) and the mitochondrial proteins arachidonate metabolism enzyme Mgst3 (glutathione S‐transferase 3), Slc25a20 (carnitine/acylcarnitine carrier), Acadsb (short/branched chain specific acyl‐CoA dehydrogenase) and Acadm (medium‐chain specific acyl‐CoA dehydrogenase). The carrier Slc25a20 is involved in the import of fatty acids of different chain lengths in the form of acylcarnitines into the mitochondrial matrix [34], [S36]. The importance of this carrier is highlighted by the observation that mutations in *SLC25A20* cause carnitine‐acylcarnitine translocase deficiency associated with an abnormal acylcarnitine profile and skeletal muscle damage [35], [S37]. The down‐regulation of Slc25a20, Acadsb and Acadm provides a mechanistic link to the elevated blood acylcarnitine levels in homozygous mice. Together with previous studies on secondary mitochondrial dysfunction in desminopathies [27, 28, 36], [S38], our new results provide a more detailed insight into the mutant desmin‐induced down‐regulation of mitochondria‐related key candidates promoting metabolic dysregulation in skeletal muscle tissue.

Given the abundance of pathological protein aggregates in both hetero‐ and homozygous R405W desmin knock‐in mice, changes in the regulation of candidates related to protein quality control could be anticipated. Indeed, in addition to several E3 ubiquitin‐protein processing proteins (Klhl21, Klhl38, Maea, March7, Rmnd5a, Sh3rf2, Trim35, Uchl1, Ufc1), our analysis depicted increased levels of autophagy‐related (Sqstm1/p62, Atg101, Ulk1) and proteasome‐related proteins (Gan, Usp2), but no overexpression of small heat shock proteins (HspB1/hsp27, HspB5/αB‐crystallin), desmin and filamin‐C, all of which have previously been described to be enriched in protein aggregates [24]. Out of the known list of myofibrillar myopathy–related genes [23], only the protein quality control related candidates Dnajb6 and Bag3 were significantly up‐regulated in soleus muscle from both hetero‐ and homozygous R405W desmin mice at three months of age. Since our analysis also depicted markedly augmented levels of the transcription factors Mlf1 and Mlf2 (myeloid leukaemia factors 1 and 2), which have been reported to bind to Dnajb6 (Mlf1 and Mlf2) [34], [S39] and Bag3 (Mlf2) [34], the upregulation of this trio of proteins is of particular interest in the early disease stages. Notably, Mlf2, an interaction partner of Mlf1 [37], has been shown be enriched in protein aggregates in human desminopathies [24] thus highlighting a novel and conceivable disease link between Mlf1 and Mlf2 related transcriptional control and Dnajb6 and Bag3 mediated protein quality control.

### 4.4 Effects of the Expression of R405W Mutant Desmin on Desmin Binding Partners, Intermediate Filament Proteins and the Myofibrillar Damage Marker Xin

When addressing possible effects of the R405W mutant desmin on the expression levels of known direct desmin interaction partners ((Table S8, 2nd sheet, column Q), [1, 22], [S27]), solely the expression of the intermediate filament protein syncoilin was increased in hetero‐ and homozygous mice, whereas vimentin, plectin, nesprin‐3, spectrin alpha chain 1, ankyrin‐1, Hspb1/Hsp27, HspB5/Cryab/αB‐crystallin, myomesin‐1, myotubularin, calpain‐1, calpain‐3 and caspase‐6 remained unchanged. Lamin‐B2, synemin and stromal interaction molecule 1 were up‐ and nestin down‐regulated, however, only in the homozygous genotype. Out of the group of intermediate filament proteins, keratin‐19 was found to be down‐regulated in the soleus muscle from hetero‐ and homozygous R405W desmin knock‐in mice. This is of interest, as the absence of Krt19 in mice has been demonstrated to cause skeletal myopathy with mitochondrial and sarcolemmal reorganization [38]. In relation to the presence of marked myofibrillar damage on the ultrastructural level, our analysis also showed a significant up‐regulation of Xin/Xirp2 on the protein level in both R405W desmin genotypes. Xin along with filamin‐C has been described as sensitive marker for small and larger areas of myofibrillar lesions due to a rapid enrichment of both proteins to segments of sarcomeric damage at the level of Z‐discs [39, 40], [S40, S41].

## 5 Conclusions

Our study demonstrates that the expression of R405W mutant desmin leads to a myofibrillar myopathy in hetero‐ and homozygous R405W desmin knock‐in mice. The results from heterozygous mice further highlight the notion that the presence of desmin‐positive protein aggregates is not necessarily associated with muscle weakness. In line with previous results from the analysis of desmin knock‐out mice, measurement of blood acylcarnitine levels appears to be of potential clinical interest in the context of recessive desminopathies. Out of the known desmin binding partners, only the expression of syncoilin was increased. With regard to myofibrillar damage, the lesion marker Xin was up‐regulated in R405W desmin knock‐in mice. Our data delineate a pathogenetic link between the myeloid leukaemia factors 1 and 2 and the myofibrillar myopathy– and protein quality control‐related proteins Dnajb6 and Bag3 in the context of desminopathies. Our main finding is that extensive secondary mitochondrial damage, including dysfunction related to proton gradient‐dependent carriers, is an important early step in the pathogenesis of R405W/R406W and R349P/R350P desminopathies, and these processes are therefore potential pre‐clinical therapeutic targets. Integrated morphological, transcriptomic and proteomic data analysis is a powerful approach to identify and validate early stages of disease in desminopathies and other forms of myofibrillar myopathies.

## Conflicts of Interest

The authors declare no conflicts of interest. R.S. and C.S.C. serve as consultants for MIRA Vision Microscopy GmbH.

## Ethical Statement

Mice were handled in accordance with European Union guidelines and French regulations for animal experimentation, and the investigations were approved by the University Paris Diderot local committee (authorization number CEB‐16‐2016/2016041216476300).

## Supporting information


**Table S1:** **Primer pairs for quantitative real‐time PCR**. Primers used were designed using NCBI BLAST, Primer3plus (https://www.primer3plus.com/index.html) or OriGene (URL no longer available).

**Table S2:** **Results of the transcriptome analysis**. RNA sequencing data set from soleus muscle from heterozygous and homozygous R405W desminopathy mice and wild‐type littermates.

**Table S3:** **Transcriptomics Flame analysis**. Functional enrichment analysis of the significantly regulated mRNAs with > 1.4‐fold regulation (Table S2, column G [HOM vs. WT]) using the Flame software (https://pavlopoulos‐lab‐services.org/shiny/app/flame). Enrichment results from multiple runs are presented in the ‘Combination’ tab or in separated tabs for each tool.

**Table S4:** **Subset of transcriptomic analysis results**. Subset of protein‐coding RNAs (*p* < 0.01, >2‐fold regulation) in homozygous and wild‐type soleus muscle as shown in the volcano plot (Figure 6d). mRNAs that showed up‐ or down‐regulation that was shared between the heterozygous and homozygous genotypes are in bold orange or blue and are also listed on a separate sheet.

**Table S5:** **Results of the proteome analysis**. Data set from proteotypic peptide‐based quantitative proteomic analysis using soleus muscle tissue derived from hetero‐ and homozygous R405W desminopathy mice and wild‐type littermates. The table contains several additional sheets with subsets of data as described in the Results section.

**Table S6:** **Proteomics Flame analysis**. Functional enrichment analysis of the 638 significantly regulated proteins (Figure 7b, Table S5) using the Flame software (https://pavlopoulos‐lab‐services.org/shiny/app/flame). Enrichment results from multiple runs are presented in the ‘Combination’ tab or in separated tabs for each tool.

**Table S7:** **Correlation between transcriptome and proteome analysis results**. Correlation analysis of the combined transcriptomic and proteomic datasets with a significance level of *q* < 0.05 set for the mRNA and corresponding protein list entries resulted in four different groups of regulation; colour coding as in the dot plot visualization (Figure S3).

**Table S8:** **Detailed comparison of the transcriptome and proteome datasets**. Comparison, as described in the Results section, of the 4230 entries from the combined transcriptomic and proteomic datasets. Candidates of interest were sorted into the indicated categories. Below these categories, all remaining candidates from that dataset are listed in alphabetical order. A subset of candidates that have been identified as direct desmin interaction partners, protein aggregate myopathy causing genes, or were reported to be enriched in fibres containing sarcoplasmic protein aggregates in skeletal muscle tissue from desminopathy patients are also summarized on a separate sheet.

**Figure S1:** **Histological characterization of the skeletal muscle pathology in R405W desmin knock‐in mice**. (**a–f**) Haematoxylin and eosin (H&E) stained transverse cryosections of soleus muscle derived from 3‐month‐old (a–c, f) and 15‐month‐old (d, e) hetero‐ and homozygous knock‐in mice and wild‐type littermates. Note that homozygous animals have a markedly reduced life span limited to 3–4 months due to a lethal intestinal pseudo‐obstruction [1]. A singular, but consistent pattern in young and aged heterozygous soleus muscle was an increase in the number of internalized myonuclei (arrowheads). Homozygous mice displayed a myopathic pattern with an increase of endomysial connective tissue (arrows pointing towards each other), increased fibre size variability, atrophic muscle fibres (arrows) and an increased number centralized myonuclei (arrowheads). (**g–i**) Collagen I immunostaining revealed increased staining intensity and broadening of the endomysium in the homozygous sample. Images (g–i) were derived from serial cryosections of the samples in (a–c), with corresponding fields of view selected. (**j–l**) Myosin heavy chain slow isoform (MHCs) staining of serial cryosections of the samples in (a–c) showed no significant differences in the fraction of type 1 fibres between the three genotypes.

**Figure S2:** **Protein aggregation pathology in skeletal muscle of heterozygous and homozygous R405W desmin knock‐in mice**. (**a, b**) Subsarcolemmal protein aggregates (G) and mitochondrial accumulation (M) in soleus muscle of heterozygous animals. Note the additional presence of a large vacuolar structure (A) in (b). (**c**) Cytoplasmic body (C) adjacent to a myonucleus (N) and surrounded by mitochondria (M). (**d**) Intermyofibrillar protein aggregate (G) composed of predominantly unstructured, granular material. (**e**) Subsarcolemmal protein aggregate (G) and mitochondrial accumulation (M) in soleus muscle of a homozygous animal. (**f**) Subsarcolemmal protein aggregates (G) containing electron dense material and abnormally shaped and enlarged mitochondria (M). (**g**) Filamentous protein aggregate (G) in close proximity to a myonucleus (N), mitochondria (M) and a myofibril. (**h**) Electron dense protein aggregate (G) in the intermyofibrillar space at the level of two adjacent Z‐discs and abnormally shaped and enlarged mitochondria (M).

**Figure S3:** **Correlation of transcriptomic and proteomic data**. (**a**) Dot plot and correlation analysis of the transcriptomic and proteomic datasets using only entries that were unique and present in both data sets. The level of significance was set to *q* < 0.05 (corrected *p* value) for both mRNAs and proteins. X‐axis, log_2_‐transformed mean difference (hom/wt) of mRNA abundance (fold change); Y‐axis, log_2_‐transformed mean difference (hom/wt) of protein abundance (fold change). A linear model was used to analyse correlation and resulted in LM AdjRsquared = 0.041 with a statistical significance of *p* = 0.003. Orange dots represent genes upregulated on both mRNA and protein levels (*n* = 32), blue dots represent genes downregulated on both mRNA and protein levels (*n* = 90), yellow dots represent genes upregulated on mRNA but downregulated on protein level (*n* = 13), and green dots represent genes downregulated on mRNA but upregulated on protein level (*n* = 52).

**Data S1:** Supplementary Information.
