# Supplementary material for: R405W Desmin Knock‐In Mice Highlight Alterations of Mitochondria, Protein Quality Control and Myofibrils in Myofibrillar Myopathy
Source: J Cachexia Sarcopenia Muscle. 2025 Oct 30;16(6):e70094. doi: 10.1002/jcsm.70094 (PMC12572951; doi:10.1002/jcsm.70094)
Supplement: Supplementary file 1 — Table S1: Primer pairs for quantitative real‐time PCR. Primers used were designed using NCBI BLAST, Primer3plus (https://www.primer3plus.com/index.html) or OriGene (URL no longer available). [file JCSM-16-e70094-s005.docx]

| **Gene** | **Forward primer** | **Reverse primer** |
| --- | --- | --- |
| *Ywhaz* | AGACGGAAGGTGCTGAGAAA | GAAGCATTGGGGATCAAGAA |
| *Rpl19* | GGGCAGGCATATGGGCATA | GGCGGTCAATCTTCTTGGATT |
| *Vim* | CGGAAAGTGGAATCCTTGCAGG | AGCAGTGAGGTCAGGCTTGGAA |
| *MyoD* | ATGGCATGATGGATTACAGCGGCC | GCTCCACTATGCTGGACAGGCAG |
| *Hspb1 (Hsp27)* | ATAGAGACCTGAAGCACCGC | CGGTCATGTTCTTGGCTGGT |
| *Chrna1* | CTTAACCAGCCTGGTGTTCTACC | GCTCCACAATGACCAGAAGGAAC |
| *Ache* | TTCCTTCGTGCCTGTGGTAGAC | TTCCTTCGTGCCTGTGGTAGAC |
| *Des* | AGCGTGACAACCTGATAGAC | TTAAGGAACGCGATCTCCTCGTTGAGGGATTCGATT |

Supplementary Table 1
