# Supplementary figures and images for: R405W Desmin Knock‐In Mice Highlight Alterations of Mitochondria, Protein Quality Control and Myofibrils in Myofibrillar Myopathy
Source: J Cachexia Sarcopenia Muscle. 2025 Oct 30;16(6):e70094. doi: 10.1002/jcsm.70094 (PMC12572951; doi:10.1002/jcsm.70094)

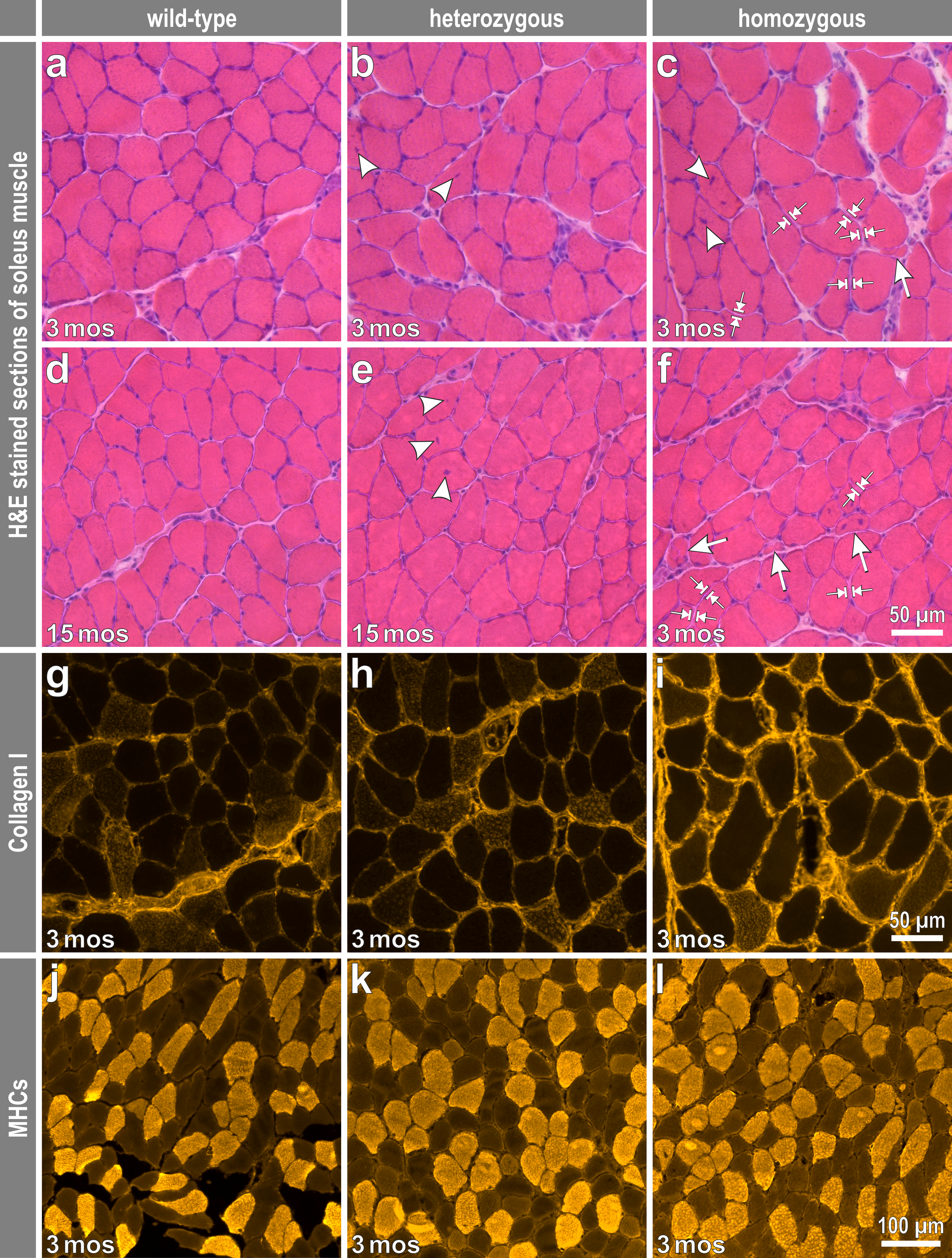

Supplement: Supplementary file 9 — Figure S1: Histological characterization of the skeletal muscle pathology in R405W desmin knock‐in mice. (a–f) Haematoxylin and eosin (H&E) stained transverse cryosections of soleus muscle derived from 3‐month‐old (a–c, f) and 15‐month‐old (d, e) hetero‐ and homozygous knock‐in mice and wild‐type littermates. Note that homozygous animals have a markedly reduced life span limited to 3–4 months due to a lethal intestinal pseudo‐obstruction [1]. A singular, but consistent pattern in young and aged heterozygous soleus muscle was an increase in the number of internalized myonuclei (arrowheads). Homozygous mice displayed a myopathic pattern with an increase of endomysial connective tissue (arrows pointing towards each other), increased fibre size variability, atrophic muscle fibres (arrows) and an increased number centralized myonuclei (arrowheads). (g–i) Collagen I immunostaining revealed increased staining intensity and broadening of the endomysium in the homozygous sample. Images (g–i) were derived from serial cryosections of the samples in (a–c), with corresponding fields of view selected. (j–l) Myosin heavy chain slow isoform (MHCs) staining of serial cryosections of the samples in (a–c) showed no significant differences in the fraction of type 1 fibres between the three genotypes. [file JCSM-16-e70094-s011.tif]

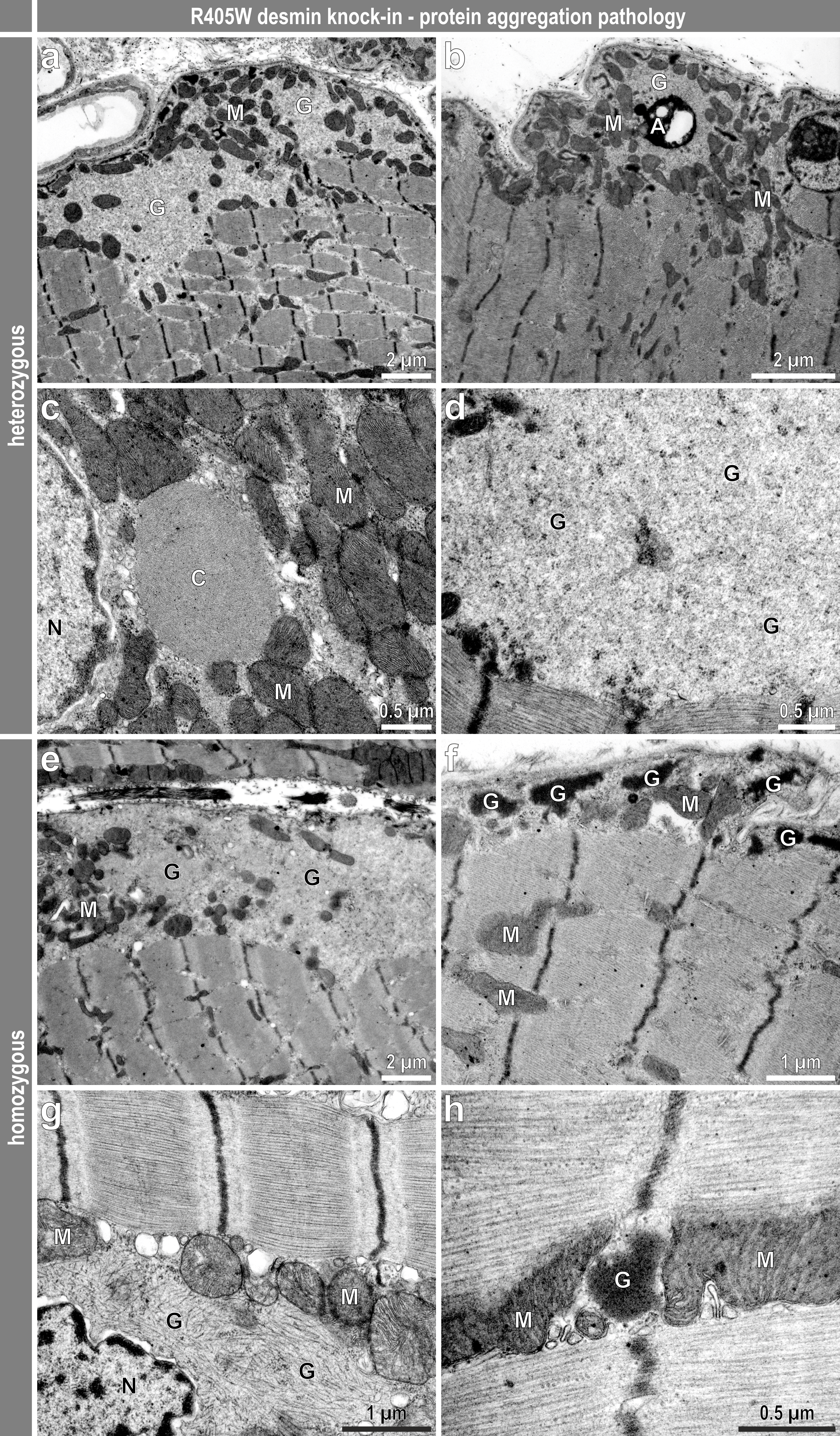

Supplement: Supplementary file 10 — Figure S2: Protein aggregation pathology in skeletal muscle of heterozygous and homozygous R405W desmin knock‐in mice. (a, b) Subsarcolemmal protein aggregates (G) and mitochondrial accumulation (M) in soleus muscle of heterozygous animals. Note the additional presence of a large vacuolar structure (A) in (b). (c) Cytoplasmic body (C) adjacent to a myonucleus (N) and surrounded by mitochondria (M). (d) Intermyofibrillar protein aggregate (G) composed of predominantly unstructured, granular material. (e) Subsarcolemmal protein aggregate (G) and mitochondrial accumulation (M) in soleus muscle of a homozygous animal. (f) Subsarcolemmal protein aggregates (G) containing electron dense material and abnormally shaped and enlarged mitochondria (M). (g) Filamentous protein aggregate (G) in close proximity to a myonucleus (N), mitochondria (M) and a myofibril. (h) Electron dense protein aggregate (G) in the intermyofibrillar space at the level of two adjacent Z‐discs and abnormally shaped and enlarged mitochondria (M). [file JCSM-16-e70094-s003.tif]

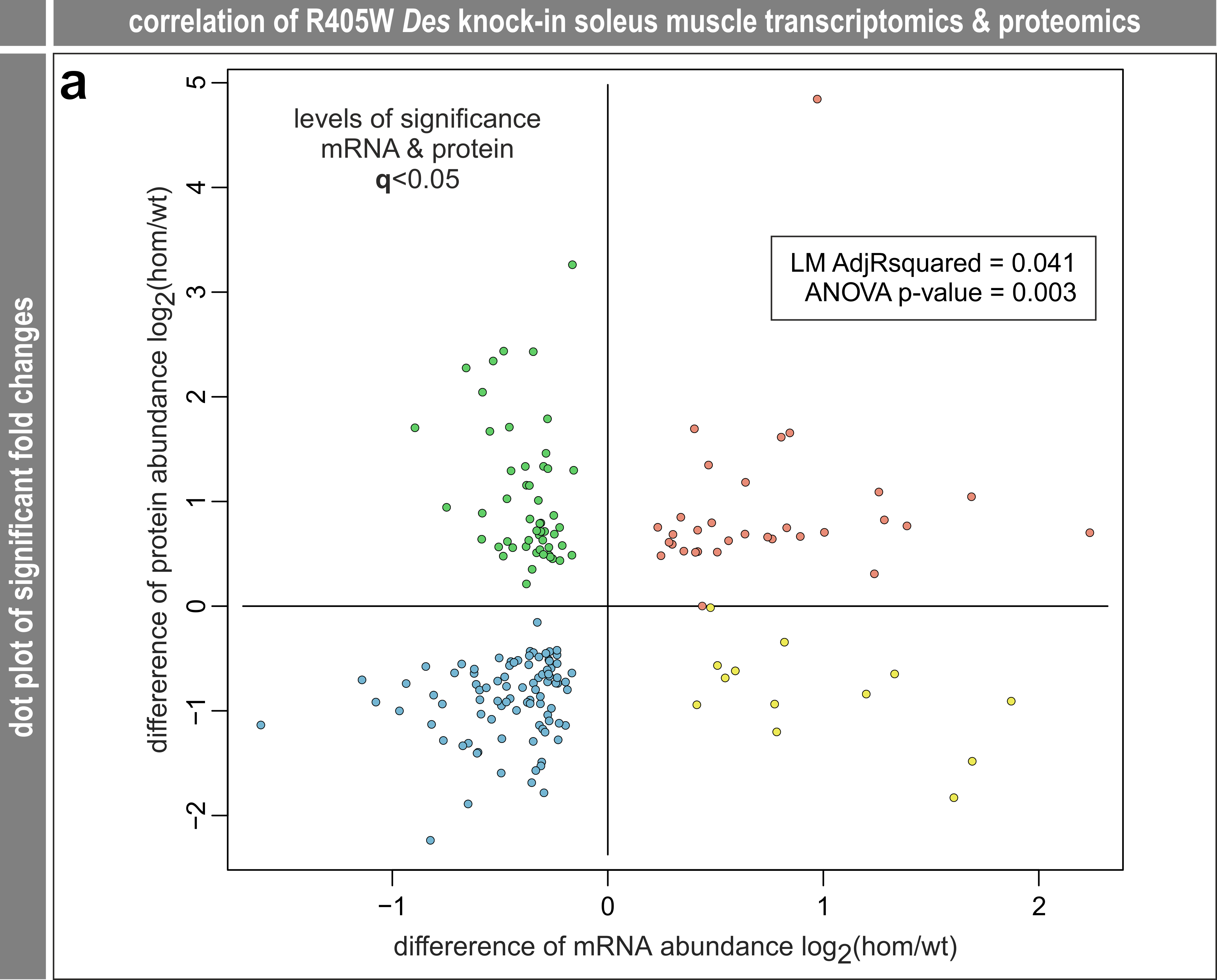

Supplement: Supplementary file 11 — Figure S3: Correlation of transcriptomic and proteomic data. (a) Dot plot and correlation analysis of the transcriptomic and proteomic datasets using only entries that were unique and present in both data sets. The level of significance was set to q < 0.05 (corrected p value) for both mRNAs and proteins. X‐axis, log2‐transformed mean difference (hom/wt) of mRNA abundance (fold change); Y‐axis, log2‐transformed mean difference (hom/wt) of protein abundance (fold change). A linear model was used to analyse correlation and resulted in LM AdjRsquared = 0.041 with a statistical significance of p = 0.003. Orange dots represent genes upregulated on both mRNA and protein levels (n = 32), blue dots represent genes downregulated on both mRNA and protein levels (n = 90), yellow dots represent genes upregulated on mRNA but downregulated on protein level (n = 13), and green dots represent genes downregulated on mRNA but upregulated on protein level (n = 52). [file JCSM-16-e70094-s001.tif]
