# Supplementary material for: R405W Desmin Knock‐In Mice Highlight Alterations of Mitochondria, Protein Quality Control and Myofibrils in Myofibrillar Myopathy
Source: J Cachexia Sarcopenia Muscle. 2025 Oct 30;16(6):e70094. doi: 10.1002/jcsm.70094 (PMC12572951; doi:10.1002/jcsm.70094)
Supplement: Supplementary file 12 — Data S1: Supplementary Information. [file JCSM-16-e70094-s006.docx]

**SUPPLEMENTAL MATERIAL**

**R405W desmin knock-in mice highlight alterations of mitochondria, protein quality control,
and myofibrils in myofibrillar myopathy**

Sabrina Batonnet-Pichon^1,2,*,#^, Florence Delort^1,*^, Alain Lilienbaum^1,*^, Carolin Berwanger^3,4^,
Dorothea Schultheis^5^, Ursula Schlötzer-Schrehardt^6^, Andreas Schmidt^7,8,ǁ^, Steffen Uebe^9^,
Yosra Baiche^1^, Tom J. Eisenack^7^, Débora Broch Trentini^7,8^, Markus Mallek^10^, Leonid Mill^11^,
Ana Ferreiro^1,12^, Bettina Eberhard^10^, Thomas Lücke^13^, Markus Krüger^7,8^, Christian Thiel^9^,
Rolf Schröder^5,§,#^, Christoph S. Clemen^3,4,§,#^

^1^ Basic and Translational Myology, Unit of Functional and Adaptive Biology, Université Paris Cité / CNRS UMR 8251, Paris, France

^2^ Institut Cochin, Université Paris Cité, INSERM U1016, CNRS, Paris, France

^3^ Institute of Aerospace Medicine, German Aerospace Center, Cologne, Germany

^4^ Institute of Vegetative Physiology, Medical Faculty, University of Cologne, Cologne, Germany

^5^ Institute of Neuropathology, University Hospital Erlangen, Friedrich-Alexander University Erlangen-Nürnberg, Erlangen, Germany

^6^ Department of Ophthalmology, University Hospital Erlangen, Friedrich-Alexander University Erlangen-Nürnberg, Erlangen, Germany

^7^ Center for Molecular Medicine Cologne (CMMC), Medical Faculty, University of Cologne, Cologne, Germany

^8^ Cologne Excellence Cluster on Cellular Stress Responses in Aging-Associated Diseases (CECAD), University of Cologne, Cologne, Germany

^9^ Institute of Human Genetics, Friedrich-Alexander University Erlangen-Nürnberg, Erlangen, Germany

^10^ MVZ Dr. Eberhard & Partner Dortmund, Dortmund, Germany

^11^ MIRA Vision Microscopy GmbH, Göppingen, Germany

^12^ Reference Center for Neuromuscular Disorders, Pitié-Salpêtrière Hospital, AP-HP, Paris, France

^13^ University Hospital of Pediatrics and Adolescent Medicine, St. Josef-Hospital, Ruhr-University Bochum, Bochum, Germany

^*^These three first authors contributed equally to this work.

^ǁ^Present address: Bruker Daltonics GmbH & Co KG, Bremen, Germany

^§^These two senior authors contributed equally to this work.

^#^Authors for correspondence:

Sabrina Batonnet-Pichon, Institut Cochin, Université Paris Cité, INSERM U1016, CNRS, F-75014 Paris, France, 75014 Paris, France; Phone: +33 1 44412423; sabrina.pichon@u-paris.fr

Rolf Schröder, Institute of Neuropathology, University Hospital Erlangen, Schwabachanlage 6, 91054 Erlangen, Germany; Phone: +49 9131 85 34782; rolf.schroeder@uk-erlangen.de

Christoph S. Clemen, Institute of Aerospace Medicine, German Aerospace Center (DLR), Linder Höhe, 51147 Cologne, Germany; Phone: +49 2203 601 3468; christoph.clemen@uni-koeln.de

**Expanded Materials and Methods**

***R405W desmin knock-in mice***

The R405W desmin knock-in mouse model C57BL/6N-*Des*^tm1.1Allb^ (http://www.informatics.jax.org/allele/MGI:6382571) was generated as described previously [1]. Routine genotyping on gDNA from tail biopsies was performed by PCR using the primer pair 5’-CTGGAGGAGGAGATCCGACA-3’ and 5’-GGCCCTCGTTAATTTTCTGC-3’. Mice were housed in isolated ventilated cages under specific and opportunistic pathogen-free (SOPF) conditions at a standard environment with free access to water and food. Health monitoring was done as recommended by the Federation of European Laboratory Animal Science Associations. Mice were handled in accordance with European Union guidelines and French regulations for animal experimentation, and the investigations were approved by the University Paris Diderot local committee (authorization number CEB-16-2016 / 2016041216476300). Moreover, the Buffon animal facility (Plateforme d’Hébergement et d’expérimentation animale Buffon) is fully licensed by French competent authorities and has animal welfare insurance.

Muscle strength was tested using a grip strength meter (Bioseb BIO-GS3+, Bioseb, Vitrolles, France) that had been re-calibrated by the manufacturer. Accurate measurements of grip strength require that the tara point is set before each new measurement, that the mice always grasp the grid of the device in the central area connected to the sensor axis, and that a mouse is slowly pulled away from the grid by its tail with almost no deviation from the horizontal orientation of the sensor axis. All measurements were conducted blinded to the genotype of the mice, in the same room in the animal facility, at the same time of day, and by the same examiner for all animals tested. Further information on the validity and limitations of grip strength measurements can be found here: a) TREAT-NMD Neuromuscular Network, SOP SMA_M.2.1.002 version 2, <https://www.treat-nmd.org/wp-content/uploads/2023/07/sma-SMA_M.2.1.002.pdf>; b) Grip Strength Tests in Rodents, Maze Engineers, 2023, <https://doi.org/10.55157/CS2023109>. For data analysis, the maximum grip strength from up to three trials until a mouse was observed to grasp the wires of the grid with both forepaws or all four paws was used.

***Histological evaluation***

Murine soleus/gastrocnemius muscle packages were dissected and immediately embedded in Tissue-Tek OCT Compound (Sakura Finetek, Torrance, CA, USA), frozen in liquid nitrogen-cooled isopentane, and stored at −80°C. Cryostat sections of 6 µm thickness were collected on microscope slides, air-dried for 30 min, and used for standard histology staining and evaluation. In addition, a deep learning-based image analysis approach using photo-realistic computer graphics generated synthetic data in muscle histopathology ([2], https://www.mira.vision) was used to quantitatively analyse the soleus muscle part in whole slide images of the hematoxylin and eosin-stained sections derived from 3-month-old mice recorded by a slide scanner (NanoZoomer S60, Hamamatsu, Japan; NDP view 2.0 digital microscopy software).

***Immunoblotting and antibodies***

For the extraction of proteins, snap-frozen soleus muscles were pulverized in a mortar on liquid nitrogen before addition of lysis buffer (50 mM TrisHCl pH 8.0, NaCl 150 mM, EDTA 1 mM, NP40 1%). Samples were rotated at 4°C for 1h and then sonicated for 10 s. Samples were not centrifugated to avoid loss of desmin, used undiluted for protein quantitation by the Bradford method, and then 1:5 diluted with 1x SDS sample buffer (25 mM Tris, 0.8% SDS, 2% 2-mercaptoethanol, 4% glycerol, 0.001% bromophenol blue, pH 6.8) and boiled at 95°C for 7 min. A total of 30 µg of muscle protein extracts were loaded per line on a 10 or 15% acrylamide gel for SDS-PAGE. After separation, proteins were transferred onto nitrocellulose membranes (0.45 µm, Macherey Nagel), which were blocked 2h with either 5% non-fat milk or 4% BSA with low endotoxin, IgG and proteases (US Biological) in 0.5% Tween/PBS solution. Membranes were incubated with polyclonal rabbit anti-p62 (SQSTM1) (P0067, Sigma-Aldrich, 1:500) for 1h at room temperature. Anti-rabbit secondary antibody coupled with horseradish peroxidase (#31460, Pierce/Thermo Scientific, 1:10,000) was detected following incubation with Clarity Western ECL (BioRad) and visualized with a CCD camera (FUJI LAS 4000, GE Healthcare).

***Mass spectrometric analysis of acylcarnitine and amino acid levels in blood***

Mass spectrometric quantitation of acylcarnitines and amino acids from murine retro-orbital sinus blood samples was performed as previously described in detail [3].

***Electron microscopy***

Soleus muscle specimens were fixed in 2.5% glutaraldehyde in 0.1 M phosphate buffer, pH 7.2, post-fixed in 2% buffered osmium tetroxide, dehydrated in graded alcohol concentrations, and embedded in epoxy resin according to standard protocols. 1 µm semi-thin sections for orientation were stained with toluidine blue. Ultra-thin sections were stained with uranyl acetate and lead citrate, and examined with a LEO 906E transmission electron microscope (Carl Zeiss GmbH, Oberkochen, Germany) at the Department of Ophthalmology, University Hospital Erlangen, Friedrich-Alexander University Erlangen-Nürnberg, Erlangen, Germany, or a JEOL 1011 transmission electron microscope (JEOL GmbH, Freising, Germany) at the Electron Microscopy Platform, Cochin Institute, Paris, France.

***Immunofluorescence microscopy and antibodies***

Muscle tissue sections were fixed for 10 min with acetone at −20°C, and air-dried for 10 min. Non-specific binding was blocked with 10% fetal calf serum, 1% goat serum, and 0.01% sodium azide in PBS for 1h at room temperature. Incubation with primary antibodies diluted in PBS with 3% BSA was done overnight at 4°C. After washing, sections were incubated with secondary antibodies, and finally washed with PBS and mounted in Mowiol. Desmin immunofluorescence images were acquired with a Leica TCS SP5 confocal laser scanning microscope equipped with a HCX PL APO 63x/1.40-0.60 Oil Lambda blue objective, HyD detectors and LAS AF software. Collagen I and myosin heavy chain (MHC) isoform immunofluorescence images were acquired using a slide scanner (NanoZoomer S60, Hamamatsu, Japan; NDP view 2.0 digital microscopy software).

The following antibodies were used: Rabbit polyclonal anti-desmin-CT1 ([1], 1:100), which can be used for detection of total human and murine desmin in tissue sections and cells; rabbit polyclonal anti-R406W-desmin ([1], 1:50), which can be used for detection of human R406W and murine R405W-desmin in tissue sections and cells; rabbit polyclonal anti-collagen I (AB765P, Sigma-Aldrich/Chemicon, 1:300); mouse monoclonal anti-myosin heavy chain slow (NCL-MHCs, Leica Biosystems/Novocastra, 1:50); mouse monoclonal anti-myosin heavy chain fast (NCL-MHCf, Leica Biosystems/Novocastra, 1:20); mouse monoclonal anti-myosin heavy chain developmental (NCL-MHCd, Leica Biosystems/Novocastra, 1:20); mouse monoclonal anti-myosin heavy chain neonatal (NCL-MHCn, Leica Biosystems/Novocastra, 1:10). Secondary antibodies were donkey anti-rabbit IgG Alexa Fluor 555 (Invitrogen, #A31572) at 1:250 dilution and goat anti-mouse IgG Alexa Fluor 555(Invitrogen, #A21424) at 1:400 dilution.

***Proteomics***

Single snap-frozen soleus muscles derived from 3-month-old mice were pulverized at −80°C and proteins were extracted using 50 µl of a 4% SDS in PBS lysis buffer combined with 10 min sonication (30 seconds on, 30 seconds off) in a Bioruptor sonication bath (Diagenode, Denville, USA) at 14°C. Samples were heat inactivated at 90°C for 10 min, centrifuged at 10,000 xg to obtain the soluble supernatant, the protein content of all samples was determined with the Pierce 660 nm absorption protein assay (ThermoFisher) using a 1:10 dilution of the original sample, and an aliquot of 20 µg total protein of each sample was placed in a new vial. Disulfide bonds were reduced and alkylated using TCEP (bond breaker, ThermoFisher) and CAA (chloroacetamide, Sigma), both 10 mM in 50 mM ammonium bicarbonate at 70°C for 20 min. Samples were diluted with 50 mM ammonium bicarbonate to reach a volume of 45 µl, magnetic SP3 protein affinity beads (2.5 µl) were added to the samples, and immediately 50 µl of acetonitrile were added. Proteins were allowed to bind for 10 min, followed by washing with 70% ethanol (3x 100 µl) and 100% acetonitrile (100 µl). After drying, proteins were digested with 0.5 µg trypsin in 50 mM ammonium bicarbonate at 35°C for 12h. Subsequently, peptide mixtures were desalted on SDB-RP stage tips, vacuum dried and redissolved in LC-MS loading buffer (5% FA, 2% ACN in water).

For LC-MS analysis, peptides were separated using the nanoElute HPLC chromatography system (Bruker Daltonics, Bremen, Germany) equipped with a 5 mm trapping column and a 25 cm separation column (Thermo-Fisher Scientific, Bremen, Germany). A linear gradient from 5 to 35% ACN was applied over a time of 35 min to fractionate the peptide sample (pulled tip, 300 nL/min, 50 min). Eluting peptides were directly ionized and detected in a timsTOF Pro2 mass spectrometer (Bruker Daltonics, Bremen, Germany) using a data-independent acquisition strategy (dia-PASEF with 12x 50 Da isolation windows in 4x 100 ms PASEF ramps). Data files were searched against the Prosit mouse database using the DIA-NN 1.8.1 software suite [4]. Search settings were fixed modification for Cys carbamidomethylation, variable oxidation of methionine side chains, sample dependent mass accuracy, double pass mode for protein identification, precursor m/z 400-1,100, fragment m/z 250-1,700, report of protein identification quality and heuristic protein inference. Protein abundances were recovered from proteotypic peptides using an in-house R-script based on the one supplied by DIA-NN [4]. Sample statistics were performed in Perseus [5]. Raw data have been deposited to the ProteomeXchange Consortium [6] (https://www.proteomexchange.org/) via the PRIDE [7] partner repository with the dataset identifier PXD042319.

***Transcriptomics***

Two snap-frozen soleus muscles per mouse, obtained from 3-month-old mice, were pulverized in a cryogenic mortar (Dominique Dutscher, Issy-les-Moulineaux, France), resuspended in buffer provided in RNeasy Fibrous Tissue Mini Kit (Qiagen, Hilden, Germany), and RNAs were extracted and eluted in RNAse free water following the manufacturer instructions. RNA integrity was controlled using RNA pico chips and the Bioanalyzer system (Agilent, Santa Clara, USA), and limit RIN number was fixed above a value of 6. RNA concentration was measured using a NanoDrop 1000 spectrophotometer (ThermoFisher Scientific, Wilmington, DE, USA). 350 ng of each sample were subjected to RNAseq (iGenSeq plateforme ICM, Paris, France), and 1 µg was used for reverse transcription (RT).

A mRNA library was prepared using the KAPA mRNA HyperPrep Kit (Roche, Basel, Switzerland) following the manufacturer’s recommendations. Final sample libraries were sequenced on Novaseq 6000 ILLUMINA with S1-200 cartridge (2x1600 Millions of 100 bases reads), corresponding to 2x32 Millions of reads per sample after demultiplexing. This work benefited from equipment and services from the iGenSeq core facility at ICM, Paris (https://igenseq.institutducerveau-icm.org/service/sequencage-arn-et-epigenetique/).

RNAseq data were analysed using RASflow_EDC v. 0.7 (https://github.com/parisepigenetics/RASflow_EDC, adapted from RASflow, [8]) using STAR [9] alignment on mm39 genome and featureCounts (with both “-M” and “—fraction” options, [10]) using NCBI RefSeq annotation (https://www.ncbi.nlm.nih.gov/assembly/GCF_000001635.27/). The complete list of parameters and tool versions is available in the repository. The RNA-Seq Analysis Snakemake Workflow (RASflow) was used to analyze raw FastQ files received from the sequencing facility, with support of Bioinformatics and Biostatistics Core Facility, Paris Epigenetics and Cell Fate Center. Differential analysis was done from raw count tables using DESeq2 [11]. The workflow was run on the HPC cluster of iPOP-UP, hosted by RPBS and funded by the Université Paris Cité (IDEX). RNAseq data from this manuscript have been deposited to the NCBI Sequence Read Archive data repository (https://www.ncbi.nlm.nih.gov/sra/) with the dataset identifier PRJNA1137105.

Additionally, RNAseq and proteomics data were jointly processed using a linear model to analyze correlation between the log_2_(fold change) values of both methods. Correlation analysis was performed using the lm() and anova() functions of the R (version 4.2.3) stats core package.

***Quantitative real-time PCR***

1 µg of each total RNA sample was used to synthetize cDNA with Transcriptor First Strand cDNA synthesis Kit (Roche) according to the manufacturer's instructions with the additional denaturation step at 65°C for 10 min. Quantitative real-time PCR was performed in duplicate using 1:20 or 1:5 dilutions of the cDNA and SYBR Green I master mix (Roche) with a LightCycler 480 II (Roche) with a denaturation step at 95°C for 8 min followed by 40 cycles of 94°C for 15 s, 55°C for 20 s and 72°C for 30 s, the melting curve was also included at a cycle of 95°C for 5 s, 60°C for 1 min and 95°C for 30 s. Six housekeeping genes were analyzed using the NormFinder Excel plugin [12], and RPL19 and YWHAZ were selected to determine relative gene expressions. Each gene expression was analyzed using the comparative ΔΔCq based on normalization with the two endogenous reference genes (*Rpl19* and *Ywhaz*) and normalized to the wild-type reference group (calibrator). The values for amplified transcripts (2^-ΔΔCq^) were plotted using GraphPad Prism v. 10 and statistics performed using the Mann-Whitney U-test. Primers used were designed using NCBI BLAST, Primer3plus (https://www.primer3plus.com/index.html) or OriGene (URL no longer available) (Table S1).

***Analysis of terminal ribosome stalling***

Reference reporter plasmids for the terminal ribosome stalling assay were a kind gift of Ramanujan Hegde (Addgene plasmids #105686 and #105688). The coding sequences of human wild-type or R406W desmin mutant were introduced into the no stall control reporter in-frame and immediately preceding the K0 linker sequence using HiFi DNA Assembly Master Mix (New England Biolabs). To create HEK293 ZNF598 knock-out cells, Flp-In T-Rex 293 cells (Invitrogen) were transiently co-transfected with the pSpCas9(BB)-2A-GFP (PX458) Cas9 expression vector [13] containing the ZNF598-targeting guide (5’-GCGCAGCTCCTCGCGGCACA-3’), and a previously described donor plasmid [14] modified to introduce a puromycin resistance gene into the targeted genomic locus. A few days after transfection, knock-out cells were selected with puromycin for 15 days. The disruption of ZNF598 was validated by immunoblotting (Anti-ZNF598 antibody, Sigma HPA041760). Flp-In T-Rex 293 WT or ZNF598 knock-out cells were transiently transfected with ribosomal stalling reporter plasmids using Lipofectamine 3000 (ThermoFisher Scientific) according to manufacturer’s instruction. 48h after transfection, cells were detached using Gibco TrypLE Express Enzyme (ThermoFisher Scientific), sedimented by centrifugation, and resuspended in ice-cold FACS buffer (3% FBS in PBS). Fluorescences of GFP and mCherry were then measured by flow cytometry in a CytoFLEX LX instrument (Beckman Coulter). Data analysis was performed using FlowJo v. 10.8.1. Intact single cells were gated using forward and side light scattering parameters. Only transfected cells were included in the analysis that were defined as cells presenting higher GFP fluorescence than control cells transfected with an empty vector.

***Miscellaneous methods***

While working on this manuscript, some of the authors found themselves in a similar unfortunate situation to that described in [15], but discovered that leisure was an effective means of self-treatment. Desmin immunofluorescence images were deconvolved using Huygens Essential version 17.10 (Scientific Volume Imaging B.V., Hilversum, The Netherlands). Grip strength graphs were generated using GraphPad Prism version 6.0.7 (GraphPad Software, Boston, MA), qRT-PCR graphs using GraphPad Prism version 10.2.3, other graphs using Excel 2016 (Microsoft) in conjunction with the add in ‘XY Chart Labeler’ version 7.1 by Rob Bovey available at http://www.appspro.com and the add-in ‘Real Statistics Resource Pack’ release 8.7 by Charles Zaiontz available at http://www.real-statistics.com, or as indicated in the respective method parts. Graphs and images were further processed and figures assembled using CorelDraw Graphics Suite X7 (Corel Corporation, Ottawa, Canada). Statistical analyses were performed as indicated in the respective Methods sections and Figure legends.

**Expanded Results**

***Homozygous R405W desmin knock-in mice displayed increased blood concentrations of acylcarnitines and amino acids***

Building on our previous work demonstrating that desmin knock-out mice harbored elevated levels of blood acylcarnitines and amino acids [3] indicative of altered muscle metabolism [16], we performed corresponding mass spectrometry analysis of dried whole blood samples derived from the R405W desmin knock-in mice. This analysis showed significantly increased concentrations of multiple acylcarnitines comprising ethyl-carnitine (acetyl-carnitine, C2), propyl-carnitine (propionyl-carnitine, C3), butyryl- and methylmalonyl-carnitine (C4, C4DC), pentyl- (isovaleryl-) and hydroxy-pentyl-carnitine (C5, C5OH), decanoyl- and cis1-decanoleyl-carnitine (C10, C10:1), hexadecanoyl- (palmitoyl-) and hydroxy-hexadecanoyl-carnitine (C16, C16OH), and octadecanoyl- and oleoyl-carnitine (C18, C18:1) in homozygous mice (Figure 1c). In addition, elevated levels of amino acids were detected, including alanine, the hydrophobic amino acids including branched valine, isoleucine and leucine, and the aromatic amino acid phenylalanine (Figure 1d).

***The R405W desmin mutation does not affect the ribosomal translation of desmin***

Our findings showed that the mono- and bi-allelic expression of the R405W mutant desmin led to a myofibrillar myopathy with desmin-positive protein aggregates and degenerative changes of the myofibrillar apparatus. To study the molecular pathophysiology of this protein aggregate myopathy in more detail, we next analysed the ribosomal translation of R405W/R406W desmin and the global, R405W desmin-induced changes on the RNA and protein levels by transcriptomics and proteomics. To evaluate putative effects of the R406W mutation on the translation of desmin, we performed a dual fluorescence assay measuring the incidence of terminal ribosomal stalling [17] (Figure 5a). Obstacles to ribosomal elongation, e.g. due to suboptimal codons, certain hard to decode peptide motifs, polybasic sequences, or mRNA damage by oxidative stress, can result in prolonged ribosomal pausing and collision of ribosomes. This leads to splitting of elongation-stalled ribosomes and co-translational degradation of the arrested nascent protein. To test if translation abortion happens during the synthesis of R406W mutant desmin, we measured how often ribosomes can read past its coding sequence, by placing it between two fluorescent proteins. The results indicated that the rates of full-length synthesis were the same for wild-type and R406W mutant desmin, and were comparable to a non-stalling control (Figure 5b). Moreover, desmin translation was not affected by the knock-out of the ribosome collision sensor ZNF598 (Figure 5c), responsible for activating the rescue of stalled ribosomes [17, 18].

***Expression of mutant desmin resulted in the detection of only 62 dysregulated mRNAs shared between hetero- and homozygous R405W desminopathy mice***

Next, RNA sequencing (RNAseq) using soleus muscle derived from hetero- and homozygous R405W desminopathy mice and wild-type littermates let to a detection of a total of 29,616 different RNA species in all three genotypes, of which 17,538 were protein coding (Table S2). A comparison of significantly (p<0.05, no fold change limit set) differentially regulated RNA species between the heterozygous and wild-type genotypes resulted in a total of 1,697 (1,389) RNAs (mRNAs), out of which 908 (762) were up- and 789 (627) were down-regulated. In the homozygous versus wild-type genotype, a total of 5,738 (5,118) RNAs (mRNAs) were regulated with 3,091 (2,761) up- and 2,647 (2,357) down-regulated (Table S2). Principal component analysis of the data derived from the five mice per genotype showed two different clusters, one with the homozygous R405W soleus samples separated from the second containing the wild-type and heterozygous genotypes (Figure 6a). Normalized counts of the top 3,582 significantly (based on the corrected p-values derived from the homozygote versus wild-type comparison) regulated RNAs were plotted in heat-map form to identify clusters of co-regulated genes among all samples. This indicated a high transcriptional heterogeneity between the homozygous and wild-type samples, while heterozygous genes displayed an intermediate transcriptional pattern with some genes close to the wild-type and others close to the homozygous condition (Figure 6b). Multiple functional enrichment analysis of the significantly regulated mRNAs contained herein with >1.4-fold regulation using the Flame software (http://flame.pavlopouloslab.info; a maximum of 3,000 entries can be analysed) primarily highlighted differentially regulated transcripts related to extracellular matrix, basement membrane, adhesion, and collagen metabolism; the KEGG pathway database confirmed this finding (Table S3). Within the limits of a significant (p<0.05) and >2-fold regulation, 1,142 (1,026 + 116) RNA species were up- and 289 (272 + 17) down-regulated in homozygous and 307 (191 + 116) up- and 127 (110 + 17) down-regulated in heterozygous soleus muscle compared to the wild-type. However, only 116 up- and 17 down-regulated RNAs were shared between heterozygous and homozygous muscles (Figure 6c). A volcano plot of differentially expressed, protein-coding RNAs (p<0.01, >2-fold regulation) in homozygous and wild-type soleus muscle illustrated 627 up-regulated mRNAs and a number of 104 down-regulated mRNA species (Figure 6d, Table S4). Out of these 731 mRNAs, only 59 and 3 mRNAs showed an up- and down-regulation, respectively, shared between the heterozygous and homozygous genotypes (Table S4, 1^st^ sheet, in bold orange and blue; Table S4, 2^nd^ sheet). These ‘double-hits’ comprised diverse mRNAs related to various cellular functions, for example, extracellular matrix (Icam4, Mmp3, Mmp13, Fsbp), reactive oxygen species metabolism (Mpv17l), smooth muscle differentiation (Olfm2), mitochondria (Cyb5r2, Smim5), and Ca^2+^/sarcoplasmic reticulum (Ryr3). Finally, we analysed and validated the mRNA levels of a few genes of interest by qRT-PCR. In line with the RNAseq results for desmin and vimentin (Figure 6e), desmin levels by qRT-PCR were similar in wild-type and homozygous R405W desmin knock-in soleus muscle (fold change desmin, qRT-PCR 0.9, RNAseq 1.0), while vimentin displayed a significant, moderate increase (fold change vimentin, qRT-PCR 1.3 with p<0.0001, RNAseq 1.4 with p<0.00624) (Figure 6f). Further candidates also showed a similar up-regulation in qRT-PCR and RNAseq (fold change MyoD, qRT-PCR 2.1 with p<0.0124, RNAseq 3.0 with p<0,0039; Chrna1, qRT-PCR 2.2 with p<0.0001, RNAseq 1.6 with p<0.0001; Ache, qRT-PCR 1.6 with p<0.0001, RNAseq 1.5 with p<0.0001) (Figure 6f, Table S2), while Hsp27 was not significantly regulated using either of the methods (fold change Hsp27, qRT-PCR 1.0, RNAseq 1.1).

***Expression of mutant desmin led to the detection of 119 dysregulated proteins shared by hetero- and homozygous R405W desminopathy mice***

We further performed a global proteomic analysis also using soleus muscle tissue derived from hetero- and homozygous R405W desminopathy mice and wild-type littermates. This analysis resulted in the detection of a total of 4,517 different proteins identified by proteotypic peptides (Table S5, 1^st^ sheet; note that the mean and median abundances are log_2_-transformed values, and abundance changes and ratios must be calculated accordingly). A comparison of significantly (q<0.05 (corrected p-values)) differentially regulated proteins between the heterozygous and wild-type genotypes resulted in a total of 209, out of which 161 were up- (more abundant) and 48 were down-regulated (less abundant), including 59 proteins which were not detected (either expressed below the detection limit of the instrument, or not expressed at all or only very weakly) in the wild-type and 5 not detected in the heterozygous condition (no fold change can be calculated for such candidates) (Table S5, 2^nd^ sheet). In the homozygous versus wild-type genotype, a total of 865 proteins had different abundances (q<0.05) with 553 being more and 312 less abundant in the homozygous genotype, including 144 proteins which were not detected in the wild-type and 17 not detected in the homozygous condition (Table S5, 3^rd^ sheet). The abundancies of desmin and vimentin showed no significant change in the proteomic analysis (Table S5). In contrast to the RNA level, the principal component analysis of the proteomic data determined main differences in the global protein expression pattern with a clear separation of all three genotypes (Figure 7a). Mean intensity values of the 638 significantly regulated proteins (based on ANOVA q<0.05 across the three genotypes (Table S5, 4^th^ sheet)) were plotted in heat-map form, which clearly separated the three genotypes (Figure 7b). Multiple functional enrichment analysis of these proteins using the Flame software (http://flame.pavlopouloslab.info) and selecting all enrichment tools primarily highlighted regulated proteins linked to ubiquitin- and proteasome-related protein quality control, mitochondria and energy metabolism, sarcoplasm, and a spectrum of metabolic processes (Table S6). In the Venn diagram of significantly (p<0.05) and >2-fold regulated proteins, 267 (169 + 98) proteins were up- and 74 (60 + 14) down-regulated in homozygous and 104 (98 + 6) up- and 25 (14 + 11) down-regulated in heterozygous soleus muscle compared to the wild-type, and the number of up-regulated proteins in both heterozygous and homozygous soleus muscle (n=98) clearly exceeded the number of down-regulated proteins (n=14) (Figure 7c). Volcano plots of differentially expressed proteins (ANOVA q<0.05, >2-fold regulation) illustrated 60 and 127 up- and 35 and 65 down-regulated proteins in the hetero- and homozygous conditions, respectively, compared to wild-type (Figure 7d,e; Table S5, 5^th^ and 6^th^ sheet). Out of the significantly regulated proteins (q<0.05 for het vs. wt and for hom vs. wt single comparisons, >2-fold regulation), 46 were up-regulated and 16 were down-regulated ‘double-hits’ shared between the heterozygous and homozygous genotypes. Moreover, also with significance (q<0.05) but without a fold change, 55 additional proteins were expressed in both hetero- and homozygous conditions but not in the wild-type, and a further 2 proteins were expressed in the wild-type but not in the hetero- or homozygous conditions (Table S5, the 119 entries in bold, and Table S5, 7^th^ sheet). The latter two proteins not detected in both desminopathy genotypes were Tep1 (telomerase protein component 1) and Mrpl33 (mitochondrial 39S ribosomal protein L33, large ribosomal subunit protein bL33m). The 16 double-down-regulated proteins comprised, for example, the intermediate filament proteins Krt19 and Syne2, the cytoskeletal proteins Macf1, Myh10, and Obscn, the mitochondrial proteins Slc25a4 (ADP/ATP translocase 1) and Marc2, and Slc27a1 (long-chain fatty acid transport protein 1) (Table S5, 7^th^ sheet, lower part). The 101 double-upregulated/expressed proteins (46 + 55) (Table S5, 7^th^ sheet, upper part) comprised, for example, the intermediate filament protein Sync, multiple proteins of the quality control such as Atg101, Bag3, Dnajb6, Gan, Klhl21, Klhl38, Ulk1, Usp2, including several E3 ubiquitin-protein processing proteins (Maea, March7, Rmnd5a, Sh3rf2, Trim35, Uchl1, Ufc1), Nup133 (nuclear pore complex protein), the mitochondrial proteins Atp5mc1 and Cox7b, Casq1 (calsequestrin-1), and the transcription factors Mlf1 and Mlf2 (myeloid leukemia factors 1 and 2).

As the electron micrographs showed multiple large vacuolar structures in heterozygous and more pronounced in homozygous soleus muscle, we complemented the proteomic analysis with quantitative immunoblotting of p62/sequestosome-1, which is a main marker of the autophagic pathway. Since the soleus muscles were used up for transcriptomics and proteomics, we used total protein extracts from gastrocnemius muscles of the same mice for immunoblotting. We detected a significant increase of p62/sequestosome-1 (1.6-fold, hom vs. wt, p=0.005; samples from 10 mice of each genotype were analysed in triplicate by Western blotting) in homozygous R405W desmin knock-in mice (Figure 7f). This finding is in line with the proteomic analysis, which showed a significant 1.75-fold up-regulation in homozygous soleus muscle (Table S5).

**Expanded Discussion**

***In search for new biomarkers: Increased blood concentrations of acylcarnitines and amino acids in homozygous R405W desmin knock-in mice***

Previous studies in human desminopathies as well as in desmin knock-out and in R349P desmin knock-in mice provided evidence for morphological and functional alterations of mitochondria leading to a persistent disease-contributing secondary mitochondrial pathology [3, 19-24]. In desmin knock-out mice, the mitochondrial dysfunction was associated with widespread defects in fatty acid metabolism, as indicated by a significant decrease of the expression level of the fatty acid transporter CD36 and a significant increase in multiple acylcarnitines ranging from C3 to C18 chain length in whole blood samples [3]. Analysis of the R405W desmin knock-in mice also revealed a reduction in CD36, as well as in several other enzymes related to fatty acid and acylcarnitine metabolism, as discussed further below, at both the mRNA and protein levels, in conjunction with significantly elevated levels of several acylcarnitines ranging in chain length from C2 to C18 in homozygous animals. A constellation in which all acylcarnitines are elevated resembles a multiple acyl-CoA dehydrogenase deficiency due to a deficiency of electron transfer flavoproteins or electron transfer flavoprotein-ubiquinone oxidoreductase (ETF-QO) [25, 26], which also leads to a disorder of the dehydrogenases involved in amino acid metabolism. The generally reduced beta-oxidation in this scenario is also associated with the cytoplasmic accumulation of acetyl-CoA, pyruvate and, as observed, alanine. If and to what extent these blood parameters, which are largely determined by skeletal muscle but also by cardiac and smooth muscle metabolism, can serve as biomarkers of secondary mitochondrial dysfunction in autosomal-recessive desminopathies requires and deserves further analysis and clinical validation.

***The R406W mutation does not induce terminal ribosomal stalling during desmin translation***

Many intermediate filament proteins, including nuclear lamins as well as cytosolic vimentin, follow a co-translational assembly mode based on the interaction of two nascent polypeptides translated by adjacent ribosomes [27]. It has been postulated that ribosomal pausing might coordinate this type of co-translational assembly [28]. While translational pausing may serve a physiological function in co-translational folding and trafficking, abnormally-long ribosomal stalls leading to ribosomal collisions may signal for ribosomal splitting (translation abortion), degradation of the unfinished nascent protein, mRNA decay, and stress response activation [29]. In this context, the proteomic analysis of soleus muscle from R405W desmin knock-in mice indicated a 2-fold increase in the levels of Ufc1 (ubiquitin-fold modifier-conjugating enzyme 1) in hetero- and homozygous mice and a halving of Ufsp2 (Ufm1-specific protease 2, involved in the conjugation and removal of Ufm1) in homozygous mice. Ufm1 is a ubiquitin-like protein modification conjugated to ER-localized ribosomes upon ribosome stalling, and has been implicated in the activation of ER-phagy and in the degradation of elongation-stalled nascent proteins associated with the Sec61 translocon [30-33]. This finding suggested that mutant desmin expression could potentially affect the cellular translation system and promote ribosomal stalling. To address if translation of the desmin transcript itself is affected, we used a dual fluorescence reporter assay, which demonstrated no incidence of abortive translation during the synthesis of either wild-type or R406W mutant desmin proteins. Therefore, it is plausible that the observed changes on the UFMylation machinery are due to the cytotoxic effects of the mutant desmin protein rather than an intrinsic problem with mutant desmin translation.

**Supplemental Figure Legends**

**Supplementary Figure 1.** **Histological characterization of the skeletal muscle pathology in R405W desmin knock-in mice.** (**a-f**) Hematoxylin and eosin (H&E) stained transverse cryosections of soleus muscle derived from 3-month-old (a-c, f) and 15-month-old (d, e) hetero- and homozygous knock-in mice and wild-type littermates. Note that homozygous animals have a markedly reduced life span limited to 3 to 4 months due to a lethal intestinal pseudo-obstruction [1]. A singular, but consistent pattern in young and aged heterozygous soleus muscle was an increase in the number of internalized myonuclei (arrowheads). Homozygous mice displayed a myopathic pattern with an increase of endomysial connective tissue (arrows pointing towards each other), increased fiber size variability, atrophic muscle fibers (arrows), and an increased number centralized myonuclei (arrowheads). (**g-i**) Collagen I immunostaining revealed increased staining intensity and broadening of the endomysium in the homozygous sample. Images (g-i) were derived from serial cryosections of the samples in (a-c), with corresponding fields of view selected. (**j-l**) Myosin heavy chain slow isoform (MHCs) staining of serial cryosections of the samples in (a-c) showed no significant differences in the fraction of type 1 fibers between the three genotypes.

**Supplementary Figure 2.** **Protein aggregation pathology in skeletal muscle of heterozygous and homozygous R405W desmin knock-in mice.** (**a, b**) Subsarcolemmal protein aggregates (G) and mitochondrial accumulation (M) in soleus muscle of heterozygous animals. Note the additional presence of a large vacuolar structure (A) in (b). (**c**) Cytoplasmic body (C) adjacent to a myonucleus (N) and surrounded by mitochondria (M). (**d**) Intermyofibrillar protein aggregate (G) composed of predominantly unstructured, granular material. (**e**) Subsarcolemmal protein aggregate (G) and mitochondrial accumulation (M) in soleus muscle of a homozygous animal. (**f**) Subsarcolemmal protein aggregates (G) containing electron dense material and abnormally shaped and enlarged mitochondria (M). (**g**) Filamentous protein aggregate (G) in close proximity to a myonucleus (N), mitochondria (M) and a myofibril. (**h**) Electron dense protein aggregate (G) in the intermyofibrillar space at the level of two adjacent Z-discs and abnormally shaped and enlarged mitochondria (M).

**Supplementary Figure 3.** **Correlation of transcriptomic and proteomic data.** (**a**) Dot plot and correlation analysis of the transcriptomic and proteomic datasets using only entries that were unique and present in both data sets. The level of significance was set to q<0.05 (corrected p-value) for both mRNAs and proteins. X-axis, log_2_-transformed mean difference (hom/wt) of mRNA abundance (fold change); Y-axis, log_2_-transformed mean difference (hom/wt) of protein abundance (fold change). A linear model was used to analyse correlation and resulted in LM AdjRsquared=0.041 with a statistical significance of p=0.003. Orange dots represent genes upregulated on both mRNA and protein levels (n=32), blue dots represent genes downregulated on both mRNA and protein levels (n=90), yellow dots represent genes upregulated on mRNA but downregulated on protein level (n=13), and green dots represent genes downregulated on mRNA but upregulated on protein level (n=52).

**Supplemental Table Legends**

**Supplementary Table 1. Primer pairs for quantitative real-time PCR.** Primers used were designed using NCBI BLAST, Primer3plus (https://www.primer3plus.com/index.html) or OriGene (URL no longer available).

**Supplementary Table 2. Results of the transcriptome analysis.** RNA sequencing data set from soleus muscle from heterozygous and homozygous R405W desminopathy mice and wild-type littermates.

**Supplementary Table 3. Transcriptomics Flame analysis.** Functional enrichment analysis of the significantly regulated mRNAs with >1.4-fold regulation (Table S2, column G (HOM vs. WT)) using the Flame software (https://pavlopoulos-lab-services.org/shiny/app/flame). Enrichment results from multiple runs are presented in the ‘Combination’ tab or in separated tabs for each tool.

**Supplementary Table 4. Subset of transcriptomic analysis results.** Subset of protein-coding RNAs (p<0.01, >2-fold regulation) in homozygous and wild-type soleus muscle as shown in the volcano plot (Figure 6d). mRNAs that showed up- or down-regulation that was shared between the heterozygous and homozygous genotypes are in bold orange or blue and are also listed on a separate sheet.

**Supplementary Table 5. Results of the proteome analysis.** Data set from proteotypic peptide-based quantitative proteomic analysis using soleus muscle tissue derived from hetero- and homozygous R405W desminopathy mice and wild-type littermates. The table contains several additional sheets with subsets of data as described in the Results section.

**Supplementary Table 6. Proteomics Flame analysis.** Functional enrichment analysis of the 638 significantly regulated proteins (Figure 7b, Table S5) using the Flame software (https://pavlopoulos-lab-services.org/shiny/app/flame). Enrichment results from multiple runs are presented in the ‘Combination’ tab or in separated tabs for each tool.

**Supplementary Table 7. Correlation between transcriptome and proteome analysis results.** Correlation analysis of the combined transcriptomic and proteomic datasets with a significance level of q<0.05 set for the mRNA and corresponding protein list entries resulted in four different groups of regulation; color coding as in the dot plot visualization (Figure S3).

**Supplementary Table 8. Detailed comparison of the transcriptome and proteome datasets.** Comparison, as described in the Results section, of the 4,230 entries from the combined transcriptomic and proteomic datasets. Candidates of interest were sorted into the indicated categories. Below these categories, all remaining candidates from that dataset are listed in alphabetical order. A subset of candidates that have been identified as direct desmin interaction partners, protein aggregate myopathy causing genes, or were reported to be enriched in fibers containing sarcoplasmic protein aggregates in skeletal muscle tissue from desminopathy patients are also summarised on a separate sheet.

**Supplemental References – Part A**

The references listed here refer to this Supplemental Material document.

1. Herrmann H, Cabet E, Chevalier NR, Moosmann J, Schultheis D, Haas J, et al. Dual functional states of R406W-desmin assembly complexes cause cardiomyopathy with severe intercalated disc derangement in humans and in knock-in mice. *Circulation* 2020;**142**:2155-71.

2. Mill L, Aust O, Ackermann JA, Burger P, Pascual M, Palumbo-Zerr K, et al. Deep learning-based image analysis in muscle histopathology using photo-realistic synthetic data. *Commun Med (Lond)* 2025;**5**:64.

3. Elsnicova B, Hornikova D, Tibenska V, Kolar D, Tlapakova T, Schmid B, et al. Desmin Knock-Out Cardiomyopathy: A Heart on the Verge of Metabolic Crisis. *Int J Mol Sci* 2022;**23**:12020.

4. Demichev V, Messner CB, Vernardis SI, Lilley KS, Ralser M. DIA-NN: neural networks and interference correction enable deep proteome coverage in high throughput. *Nat Methods* 2020;**17**:41-4.

5. Tyanova S, Temu T, Sinitcyn P, Carlson A, Hein MY, Geiger T, et al. The Perseus computational platform for comprehensive analysis of (prote)omics data. *Nat Methods* 2016;**13**:731-40.

6. Deutsch EW, Bandeira N, Perez-Riverol Y, Sharma V, Carver JJ, Mendoza L, et al. The ProteomeXchange consortium at 10 years: 2023 update. *Nucleic Acids Res* 2023;**51**:D1539-D48.

7. Perez-Riverol Y, Bai J, Bandla C, Garcia-Seisdedos D, Hewapathirana S, Kamatchinathan S, et al. The PRIDE database resources in 2022: a hub for mass spectrometry-based proteomics evidences. *Nucleic Acids Res* 2022;**50**:D543-D52.

8. Zhang X, Jonassen I. RASflow: an RNA-Seq analysis workflow with Snakemake. *BMC Bioinformatics* 2020;**21**:110.

9. Dobin A, Davis CA, Schlesinger F, Drenkow J, Zaleski C, Jha S, et al. STAR: ultrafast universal RNA-seq aligner. *Bioinformatics* 2013;**29**:15-21.

10. Liao Y, Smyth GK, Shi W. The R package Rsubread is easier, faster, cheaper and better for alignment and quantification of RNA sequencing reads. *Nucleic Acids Res* 2019;**47**:e47.

11. Love MI, Huber W, Anders S. Moderated estimation of fold change and dispersion for RNA-seq data with DESeq2. *Genome biology* 2014;**15**:550.

12. Andersen CL, Jensen JL, Orntoft TF. Normalization of real-time quantitative reverse transcription-PCR data: a model-based variance estimation approach to identify genes suited for normalization, applied to bladder and colon cancer data sets. *Cancer Res* 2004;**64**:5245-50.

13. Ran FA, Hsu PD, Wright J, Agarwala V, Scott DA, Zhang F. Genome engineering using the CRISPR-Cas9 system. *Nat Protoc* 2013;**8**:2281-308.

14. Lackner DH, Carré A, Guzzardo PM, Banning C, Mangena R, Henley T, et al. A generic strategy for CRISPR-Cas9-mediated gene tagging. *Nat Commun* 2015;**6**:10237.

15. Upper D. The unsuccessful self-treatment of a case of "writer's block". *J Appl Behav Anal* 1974;**7**:497.

16. Dambrova M, Makrecka-Kuka M, Kuka J, Vilskersts R, Nordberg D, Attwood MM, et al. Acylcarnitines: Nomenclature, Biomarkers, Therapeutic Potential, Drug Targets, and Clinical Trials. *Pharmacol Rev* 2022;**74**:506-51.

17. Juszkiewicz S, Hegde RS. Initiation of Quality Control during Poly(A) Translation Requires Site-Specific Ribosome Ubiquitination. *Mol Cell* 2017;**65**:743-50 e4.

18. Sundaramoorthy E, Leonard M, Mak R, Liao J, Fulzele A, Bennett EJ. ZNF598 and RACK1 Regulate Mammalian Ribosome-Associated Quality Control Function by Mediating Regulatory 40S Ribosomal Ubiquitylation. *Mol Cell* 2017;**65**:751-60 e4.

19. Berwanger C, Terres D, Pesta D, Eggers B, Marcus K, Wittig I, et al. Immortalised murine R349P desmin knock-in myotubes exhibit a reduced proton leak and decreased ADP/ATP translocase levels in purified mitochondria. *Eur J Cell Biol* 2024;**103**:151399.

20. Hovhannisyan Y, Li Z, Callon D, Suspene R, Batoumeni V, Canette A, et al. Critical contribution of mitochondria in the development of cardiomyopathy linked to desmin mutation. *Stem Cell Res Ther* 2024;**15**:10.

21. Lindén M, Li Z, Paulin D, Gotow T, Leterrier JF. Effects of desmin gene knockout on mice heart mitochondria. *J Bioenerg Biomembr* 2001;**33**:333-41.

22. Schröder R, Goudeau B, Simon MC, Fischer D, Eggermann T, Clemen CS, et al. On noxious desmin: functional effects of a novel heterozygous desmin insertion mutation on the extrasarcomeric desmin cytoskeleton and mitochondria. *Hum Mol Genet* 2003;**12**:657-69.

23. Winter L, Wittig I, Peeva V, Eggers B, Heidler J, Chevessier F, et al. Mutant desmin substantially perturbs mitochondrial morphology, function and maintenance in skeletal muscle tissue. *Acta Neuropathol* 2016;**132**:453-73.

24. Smolina N, Khudiakov A, Knyazeva A, Zlotina A, Sukhareva K, Kondratov K, et al. Desmin mutations result in mitochondrial dysfunction regardless of their aggregation properties. *Biochim Biophys Acta Mol Basis Dis* 2020;**1866**:165745.

25. Niederwieser A, Steinmann B, Exner U, Neuheiser F, Redweik U, Wang M, et al. Multiple acyl-Co A dehydrogenation deficiency (MADD) in a boy with nonketotic hypoglycemia, hepatomegaly, muscle hypotonia and cardiomyopathy. Detection of N-isovalerylglutamic acid and its monoamide. *Helv Paediatr Acta* 1983;**38**:9-26.

26. Cornelius N, Frerman FE, Corydon TJ, Palmfeldt J, Bross P, Gregersen N, et al. Molecular mechanisms of riboflavin responsiveness in patients with ETF-QO variations and multiple acyl-CoA dehydrogenation deficiency. *Hum Mol Genet* 2012;**21**:3435-48.

27. Bertolini M, Fenzl K, Kats I, Wruck F, Tippmann F, Schmitt J, et al. Interactions between nascent proteins translated by adjacent ribosomes drive homomer assembly. *Science* 2021;**371**:57-64.

28. Panasenko OO, Somasekharan SP, Villanyi Z, Zagatti M, Bezrukov F, Rashpa R, et al. Co-translational assembly of proteasome subunits in NOT1-containing assemblysomes. *Nat Struct Mol Biol* 2019;**26**:110-20.

29. Eisenack TJ, Trentini DB. Ending a bad start: Triggers and mechanisms of co-translational protein degradation. *Front Mol Biosci* 2022;**9**:1089825.

30. Wang L, Xu Y, Rogers H, Saidi L, Noguchi CT, Li H, et al. UFMylation of RPL26 links translocation-associated quality control to endoplasmic reticulum protein homeostasis. *Cell Res* 2020;**30**:5-20.

31. Stephani M, Picchianti L, Gajic A, Beveridge R, Skarwan E, Sanchez de Medina Hernandez V, et al. A cross-kingdom conserved ER-phagy receptor maintains endoplasmic reticulum homeostasis during stress. *Elife* 2020;**9**:1-105.

32. Scavone F, Gumbin Samantha C, Da Rosa Paul A, Kopito Ron R. RPL26/uL24 UFMylation is essential for ribosome-associated quality control at the endoplasmic reticulum. *Proc Natl Acad Sci USA* 2023;**120**:e2220340120.

33. Liang JR, Lingeman E, Luong T, Ahmed S, Muhar M, Nguyen T, et al. A Genome-wide ER-phagy Screen Highlights Key Roles of Mitochondrial Metabolism and ER-Resident UFMylation. *Cell* 2020;**180**:1160-77.e20.

**Supplemental References – Part B**

The references listed here are those of 'secondary importance' (due to space limitations) and refer to the main manuscript document.

S1. Lazarides E. Intermediate filaments: a chemically heterogeneous, developmentally regulated class of proteins. *Annu Rev Biochem* 1982;**51**:219-50.

S2. O'Neill A, Williams MW, Resneck WG, Milner DJ, Capetanaki Y, Bloch RJ. Sarcolemmal organization in skeletal muscle lacking desmin: evidence for cytokeratins associated with the membrane skeleton at costameres. *Mol Biol Cell* 2002;**13**:2347-59.

S3. Tidball JG. Desmin at myotendinous junctions. *Exp Cell Res* 1992;**199**:206-12.

S4. Kartenbeck J, Franke WW, Moser JG, Stoffels U. Specific attachment of desmin filaments to desmosomal plaques in cardiac myocytes. *EMBO J* 1983;**2**:735-42.

S5. Lapouge K, Fontao L, Champliaud MF, Jaunin F, Frias MA, Favre B, et al. New insights into the molecular basis of desmoplakin- and desmin-related cardiomyopathies. *J Cell Sci* 2006;**119**:4974-85.

S6. Capetanaki Y, Bloch RJ, Kouloumenta A, Mavroidis M, Psarras S. Muscle intermediate filaments and their links to membranes and membranous organelles. *Exp Cell Res* 2007;**313**:2063-76.

S7. Dayal AA, Medvedeva NV, Nekrasova TM, Duhalin SD, Surin AK, Minin AA. Desmin Interacts Directly with Mitochondria. *Int J Mol Sci* 2020;**21**:

S8. Heffler J, Shah PP, Robison P, Phyo S, Veliz K, Uchida K, et al. A Balance Between Intermediate Filaments and Microtubules Maintains Nuclear Architecture in the Cardiomyocyte. *Circ Res* 2020;**126**:e10-e26.

S9. Lieber RL, Roberts TJ, Blemker SS, Lee SSM, Herzog W. Skeletal muscle mechanics, energetics and plasticity. *J Neuroeng Rehabil* 2017;**14**:108.

S10. Brodehl A, Gaertner-Rommel A, Milting H. Molecular insights into cardiomyopathies associated with desmin (DES) mutations. *Biophys Rev* 2018;**10**:983-1006.

S11. Carmignac V, Sharma S, Arbogast S, Fischer D, Serreri C, Serria M, et al. A homozygous desmin deletion causes an Emery-Dreifuss like recessive myopathy with desmin depletion. *Neuromuscul Disord* 2009;**19**:600.

S12. McLaughlin HM, Kelly MA, Hawley PP, Darras BT, Funke B, Picker J. Compound heterozygosity of predicted loss-of-function DES variants in a family with recessive desminopathy. *BMC Med Genet* 2013;**14**:68.

S13. Durmus H, Ayhan O, Cirak S, Deymeer F, Parman Y, Franke A, et al. Neuromuscular endplate pathology in recessive desminopathies: Lessons from man and mice. *Neurology* 2016;**87**:799-805.

S14. Batonnet-Pichon S, Behin A, Cabet E, Delort F, Vicart P, Lilienbaum A. Myofibrillar Myopathies: New Perspectives from Animal Models to Potential Therapeutic Approaches. *J Neuromuscul Dis* 2017;**4**:1-15.

S15. Fidzianska A, Kotowicz J, Sadowska M, Goudeau B, Walczak E, Vicart P, et al. A novel desmin R355P mutation causes cardiac and skeletal myopathy. *Neuromuscul Disord* 2005;**15**:525-31.

S16. Goldfarb LG, Park KY, Cervenakova L, Gorokhova S, Lee HS, Vasconcelos O, et al. Missense mutations in desmin associated with familial cardiac and skeletal myopathy. *Nat Genet* 1998;**19**:402-3.

S17. Mavroidis M, Panagopoulou P, Kostavasili I, Weisleder N, Capetanaki Y. A missense mutation in desmin tail domain linked to human dilated cardiomyopathy promotes cleavage of the head domain and abolishes its Z-disc localization. *FASEB J* 2008;**22**:3318-27.

S18. Pica EC, Kathirvel P, Pramono ZA, Lai PS, Yee WC. Characterization of a novel S13F desmin mutation associated with desmin myopathy and heart block in a Chinese family. *Neuromuscul Disord* 2008;**18**:178-82.

S19. Arbustini E, Morbini P, Grasso M, Fasani R, Verga L, Bellini O, et al. Restrictive cardiomyopathy, atrioventricular block and mild to subclinical myopathy in patients with desmin-immunoreactive material deposits. *J Am Coll Cardiol* 1998;**31**:645-53.

S20. Cetin N, Balci-Hayta B, Gundesli H, Korkusuz P, Purali N, Talim B, et al. A novel desmin mutation leading to autosomal recessive limb-girdle muscular dystrophy: distinct histopathological outcomes compared with desminopathies. *J Med Genet* 2013;**50**:437-43.

S21. Munoz-Marmol AM, Strasser G, Isamat M, Coulombe PA, Yang Y, Roca X, et al. A dysfunctional desmin mutation in a patient with severe generalized myopathy. *Proc Natl Acad Sci USA* 1998;**95**:11312-7.

S22. Schröder R, Schoser B. Myofibrillar myopathies: a clinical and myopathological guide. *Brain Pathol* 2009;**19**:483-92.

S23. Schröder R. Protein aggregate myopathies: the many faces of an expanding disease group. *Acta Neuropathol* 2013;**125**:1-2.

S24. Olive M, Goldfarb L, Moreno D, Laforet E, Dagvadorj A, Sambuughin N, et al. Desmin-related myopathy: clinical, electrophysiological, radiological, neuropathological and genetic studies. *J Neurol Sci* 2004;**219**:125-37.

S25. Park KY, Dalakas MC, Semino-Mora C, Lee HS, Litvak S, Takeda K, et al. Sporadic cardiac and skeletal myopathy caused by a de novo desmin mutation. *Clin Genet* 2000;**57**:423-9.

S26. Upper D. The unsuccessful self-treatment of a case of "writer's block". *J Appl Behav Anal* 1974;**7**:497.

S27. Hernandez DA, Bennett CM, Dunina-Barkovskaya L, Wedig T, Capetanaki Y, Herrmann H, et al. Nebulette is a powerful cytolinker organizing desmin and actin in mouse hearts. *Mol Biol Cell* 2016;**27**:3869-82.

S28. Weihl CC, Topf A, Bengoechea R, Duff J, Charlton R, Garcia SK, et al. Loss of function variants in DNAJB4 cause a myopathy with early respiratory failure. *Acta Neuropathol* 2023;**145**:127-43.

S29. Ruppert T, Heckmann MB, Rapti K, Schultheis D, Jungmann A, Katus HA, et al. AAV-mediated cardiac gene transfer of wild-type desmin in mouse models for recessive desminopathies. *Gene Ther* 2020;**27**:516-24.

S30. Diermeier S, Iberl J, Vetter K, Haug M, Pollmann C, Reischl B, et al. Early signs of architectural and biomechanical failure in isolated myofibers and immortalized myoblasts from desmin-mutant knock-in mice. *Sci Rep* 2017;**7**:1391.

S31. Winter L, Unger A, Berwanger C, Spörrer M, Türk M, Chevessier F, et al. Imbalances in protein homeostasis caused by mutant desmin. *Neuropathol Appl Neurobiol* 2019;**45**:476-94.

S32. Mishra G, Coyne LP, Chen XJ. Adenine nucleotide carrier protein dysfunction in human disease. *IUBMB life* 2023;**75**:911-25.

S33. Beckman KB, Ames BN. The free radical theory of aging matures. *Physiol Rev* 1998;**78**:547-81.

S34. Dos Santos JM, de Oliveira DS, Moreli ML, Benite-Ribeiro SA. The role of mitochondrial DNA damage at skeletal muscle oxidative stress on the development of type 2 diabetes. *Mol Cell Biochem* 2018;**449**:251-5.

S35. Brown A, Amunts A, Bai XC, Sugimoto Y, Edwards PC, Murshudov G, et al. Structure of the large ribosomal subunit from human mitochondria. *Science* 2014;**346**:718-22.

S36. De Lucas JR, Indiveri C, Tonazzi A, Perez P, Giangregorio N, Iacobazzi V, et al. Functional characterization of residues within the carnitine/acylcarnitine translocase RX2PANAAXF distinct motif. *Mol Membr Biol* 2008;**25**:152-63.

S37. Dambrova M, Makrecka-Kuka M, Kuka J, Vilskersts R, Nordberg D, Attwood MM, et al. Acylcarnitines: Nomenclature, Biomarkers, Therapeutic Potential, Drug Targets, and Clinical Trials. *Pharmacol Rev* 2022;**74**:506-51.

S38. Hovhannisyan Y, Li Z, Callon D, Suspene R, Batoumeni V, Canette A, et al. Critical contribution of mitochondria in the development of cardiomyopathy linked to desmin mutation. *Stem Cell Res Ther* 2024;**15**:10.

S39. Blandin G, Marchand S, Charton K, Daniele N, Gicquel E, Boucheteil JB, et al. A human skeletal muscle interactome centered on proteins involved in muscular dystrophies: LGMD interactome. *Skelet Muscle* 2013;**3**:3.

S40. Chevessier F, Schuld J, Orfanos Z, Plank AC, Wolf L, Maerkens A, et al. Myofibrillar instability exacerbated by acute exercise in filaminopathy. *Hum Mol Genet* 2015;**24**:7207-20.

S41. Shatunov A, Olive M, Odgerel Z, Stadelmann-Nessler C, Irlbacher K, van Landeghem F, et al. In-frame deletion in the seventh immunoglobulin-like repeat of filamin C in a family with myofibrillar myopathy. *Eur J Hum Genet* 2009;**17**:656-63.

S42. Lin YJ, Huang LH, Huang CT. Enhancement of heterologous gene expression in Flammulina velutipes using polycistronic vectors containing a viral 2A cleavage sequence. *PLoS One* 2013;**8**:e59099.

S43. Juszkiewicz S, Hegde RS. Initiation of Quality Control during Poly(A) Translation Requires Site-Specific Ribosome Ubiquitination. *Mol Cell* 2017;**65**:743-50 e4.

S44. Oliveros JC. Venny. An interactive tool for comparing lists with Venn's diagrams. 2015. https://bioinfogp.cnb.csic.es/tools/venny/index.html.
